# Cell Clustering Promotes a Metabolic Switch that Supports Metastatic Colonization

**DOI:** 10.1016/j.cmet.2026.03.008

**Published:** 2026-04-07

**Authors:** Christiaan F. Labuschagne, Eric C. Cheung, Julianna Blagih, Marie-Charlotte Domart, Karen H. Vousden

## Main text

(Cell Metabolism *30*, 720–734.e1–e5; October 1, 2019)

In the original version of Figure 4F, the probe was incorrectly labeled as BODIPY C11. As correctly described in the STAR Methods, the probe used was BODIPY C12. This mistake does not affect the main results or conclusions of the paper. The authors apologize for this error and any confusion it may have caused.

**Figure 4F:**
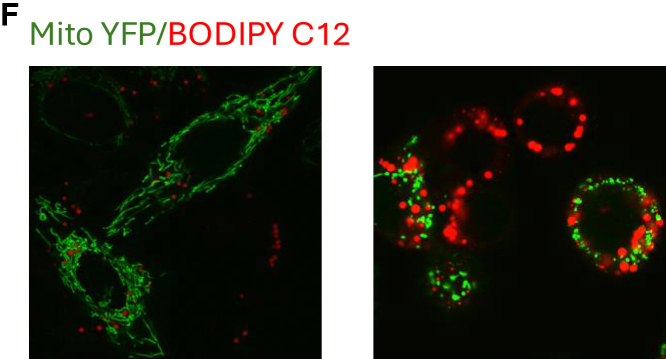
Detached Cells Are Dependent on Glycolysis for ATP Production and Survival (corrected)

