# Supplementary material for: Cell Clustering Promotes a Metabolic Switch that Supports Metastatic Colonization
Source: Cell Metab. 2019 Oct 1;30(4):720–734.e5. doi: 10.1016/j.cmet.2019.07.014 (PMC6863392; doi:10.1016/j.cmet.2019.07.014)
Supplement: Document S1. Figures S1–S7 and Table S1 [file mmc1.pdf]

Cell Metabolism, Volume *30*

Supplemental Information

Cell Clustering Promotes a Metabolic Switch that Supports Metastatic Colonization

Christiaan F. Labuschagne, Eric C. Cheung, Julianna Blagih, Marie-Charlotte Domart, and Karen H. Vousden

Supplemental Information

Figure S1

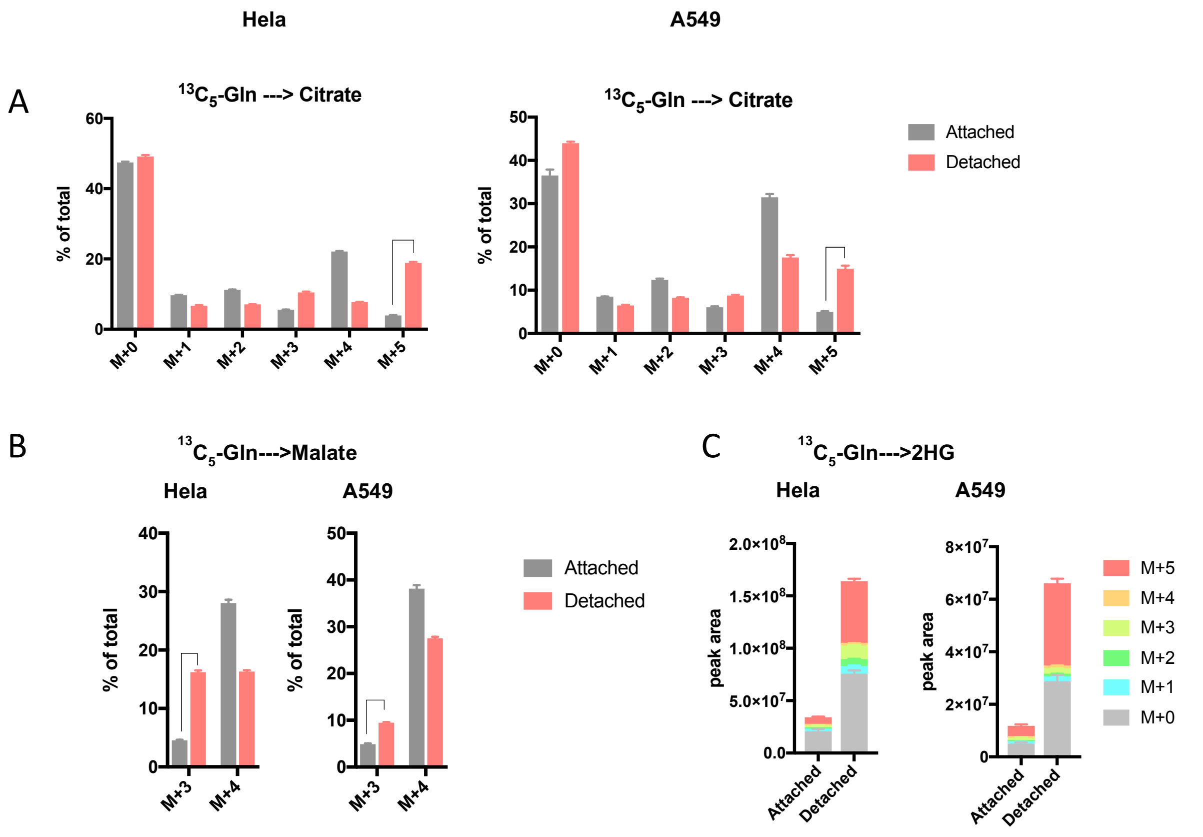

Figure S1. Related to Figure 1.

(A and B) Isotopomer distribution of (A) citrate and (B) malate in attached and detached Hela and A549 cells cultured with $^{13}C_5$-glutamine for 4 hours.

(C) Levels and isotopomer distribution of 2-hydroxyglutarate in attached and detached Hela and A549 cells cultured in the same conditions as (A). Peak area levels are normalized to cell number.

Data are presented as mean ± SD of triplicate cultures of representative experiments.

Figure S2

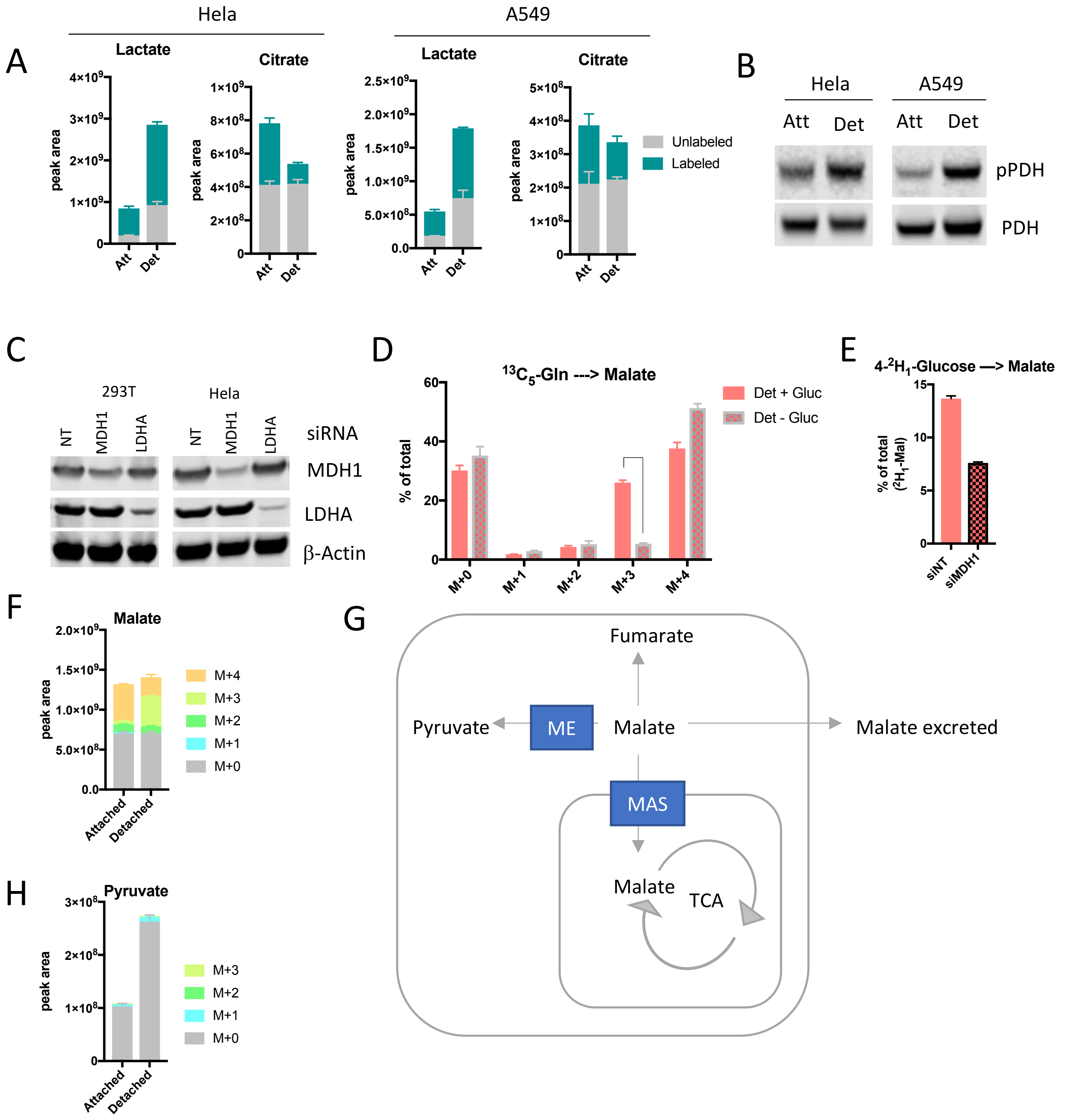

Figure S2. Related to Figure 2

(A) Levels of isotope labelling of glycolytic intermediates of Hela and A549 cells cultured in attached and detached conditions in the presence of $^{13}C_6$-glucose for 4 hours. Peak area levels were normalized to cell number.

(B) Western Blot analysis demonstrating pyruvate dehydrogenase phosphorylation in Hela and A549 attached and detached cells. Equal protein amounts were loaded per condition.

(C) Western blot analysis showing expression levels of MDH1, LDHA and β-Actin after siRNA knock down of MDH1 and LDHA in detached 293T and Hela cells.

(D) Isotopomer distribution of malate in detached 293T cells labelled with $^{13}C_5$-glutamine for 4 hours in the presence or absence of glucose.
(E) Levels of deuterium labelled malate from 4-$^2H_1$-glucose labelling in detached A549 cells following siRNA knock down of MDH1.
(F) Levels and isotopomer distribution of malate in attached and detached 293T cells labelled with $^{13}C_5$-glutamine for 4 hours.
(G) A schematic representation of the fate of cytosolic produced malate in detached cells.
(H) Levels and isotopomer distribution of pyruvate in attached and detached 293T cells labelled with $^{13}C_5$-glutamine for 4 hours.
(A, D, E, F and H) Data are presented as mean ± SD of triplicate cultures of representative experiments.

Figure S3

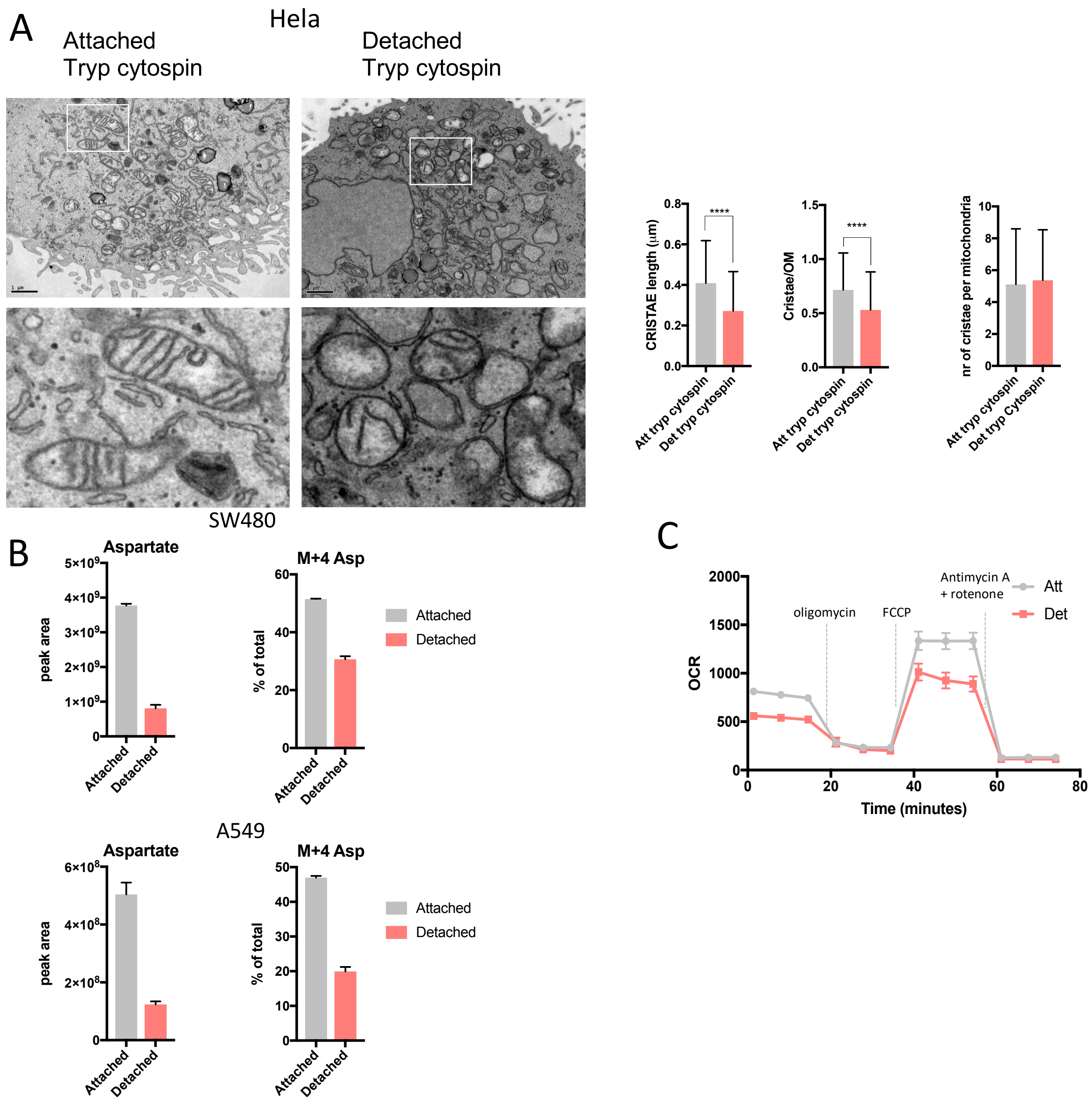

Figure S3. Related to Figure 3

(A) Representative transmission electron microscopy images of mitochondria of attached and detached cells trypsinised into single cells before cytospin to attach cells to glass bottom plates. Graphs present quantified cristae length, cristae/outer membrane ratio and number of cristae per mitochondria (Att n= 136 mitochondria and Det n=250 mitochondria). ****$p \leq 0.0001$, unpaired t test.

(B) Intracellular levels of unlabelled and M+4 labelled aspartate from $^{13}C_5$-glutamine in attached and detached SW480 and A549 cells. Peak area levels are normalized to cell number.

(C) Oxygen consumption rate profiles of SW480 cells cultured in attached or detached conditions. Dotted lines show incubation of cells in the presence of indicated mitochondrial inhibitors. Data are normalised to protein content.

(A, B and C) Data are presented as mean ± SD of triplicate cultures of representative experiments.

Figure S4

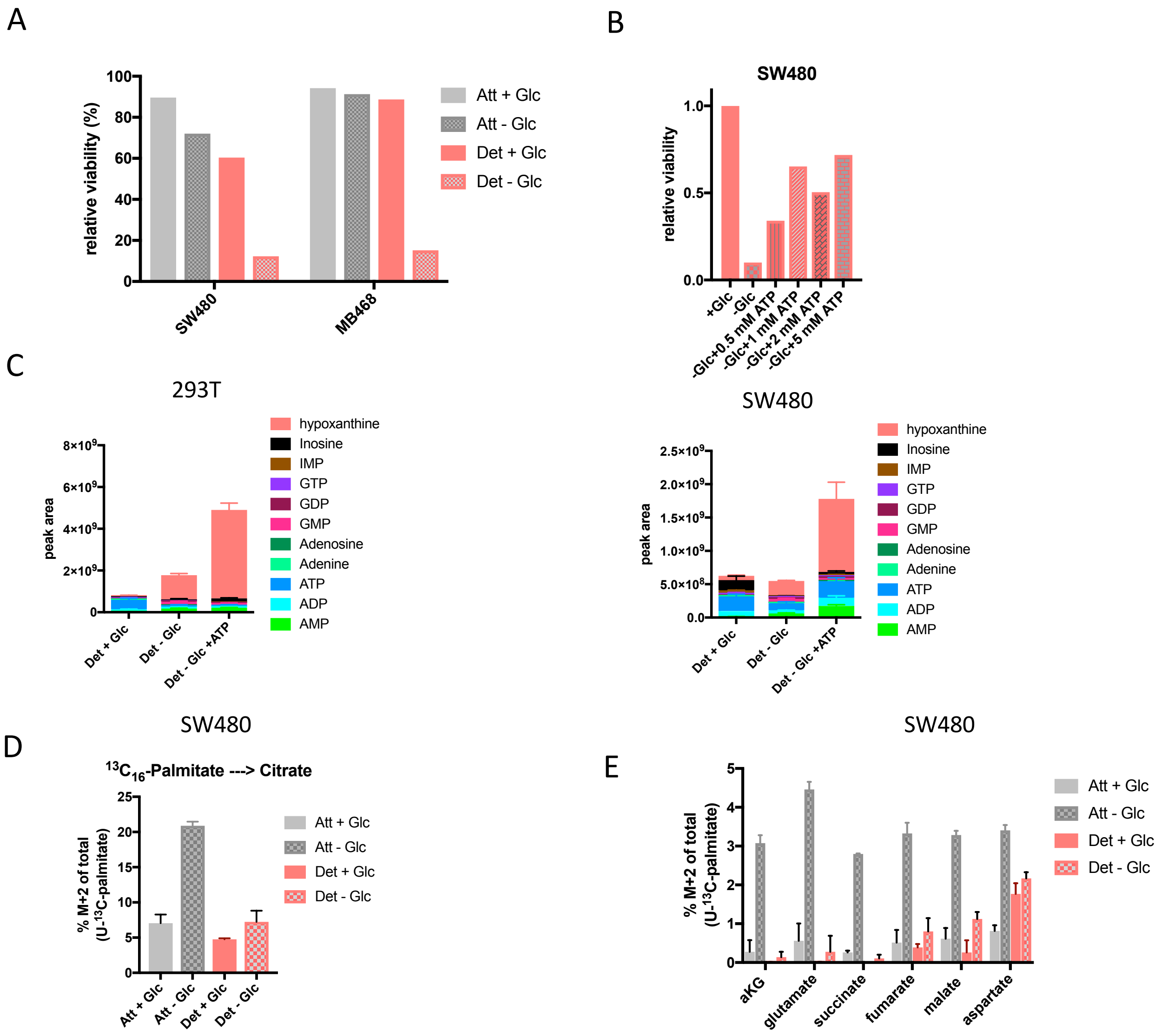

Figure S4. Related to Figure 4

(A) Cell viability of SW480 and MB468 cells cultured in attached and detached conditions in the presence or absence of glucose. Cells were cultured in attached or detached conditions for 7 days in the presence of glucose before glucose starvation for 24 hours followed by staining with eFluor 780 viability dye and analysis by flow cytometry.

(B) Cell viability of SW480 cells as measured in (A) in cells cultured with glucose or without glucose plus indicated ATP concentrations.

(C) Intracellular nucleotide levels of detached 293T and SW480 cells cultured with or without glucose plus 5 mM ATP. Peak area levels are normalised to cell number.

(D and E) Levels of M+2 citrate (D) and other TCA cycle intermediates (E) originating from $^{13}C_{16}$-palmitate in SW480 attached and detached cells cultured with or without glucose. Cells were cultured as in (A) before addition of labelled palmitate and removal of glucose for 4 hours.

(C-E) Data are presented as mean ± SD of triplicate cultures of representative experiments.

Figure S5

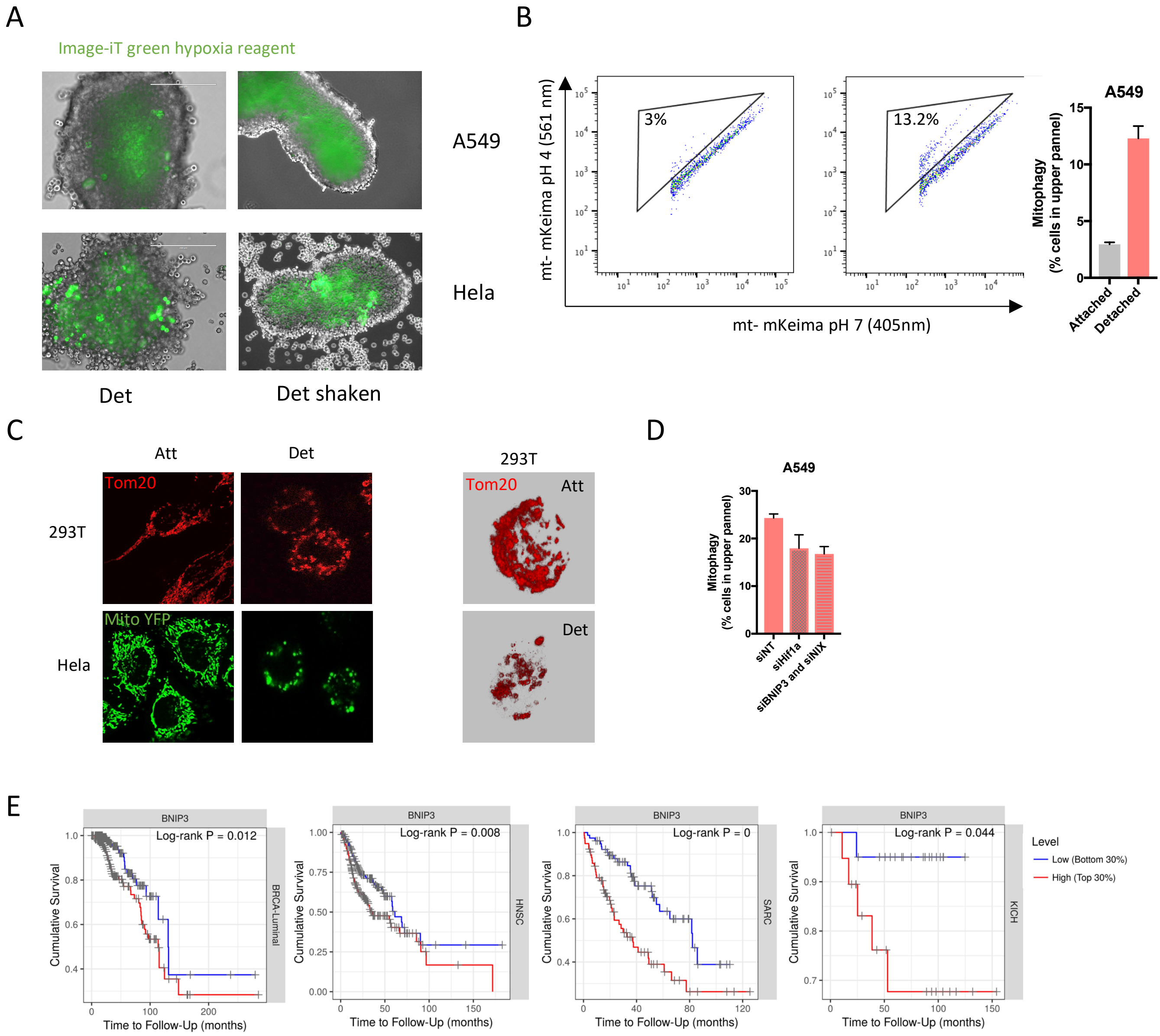

Figure S5. Related to Figure 5

(A) Image-iT green hypoxia reagent staining of detached A549 and Hela cells cultured in static (left panel) and shaken (right panel) conditions. Cells were cultured in detached conditions for 3 days before live cell staining was performed.

(B) A549 cells were transfected with mitochondrial targeted-mkeima and cultured in attached or detached conditions for 3 days before flow cytometry analysis. Mitochondria in cytosol (pH 7) are represented by mkeima with excitation of 405 nm while mitochondria in lysosomes (pH 4) are represented by mkeima with excitation of 561 nm. Mitophagy is reflected by the percentage of cells in the upper panel.

(C) Confocal microscopy images of attached and detached 293T and Hela cells. 293T cells were grown in attached or detached conditions for 7 days before cells were fixed followed by immunostaining with Tom20 antibody. Hela cells expressing Mito YFP were cultured in attached or detached conditions for 7 days before imaging.

(D) Mitochondrial targeted-mkeima expressing A549 cells were cultured in detached conditions in the presence of non-targeting, Hif1α or BNIP3 + NIX siRNA and analysed by flow cytometry for mitophagy as in (B).
(E) Patient survival data of tumors expressing high and low levels of BNIP3 in indicated cancers types.
(B and D) Data are presented as mean $\pm$ SD of triplicate cultures of a representative experiment.

Figure S6

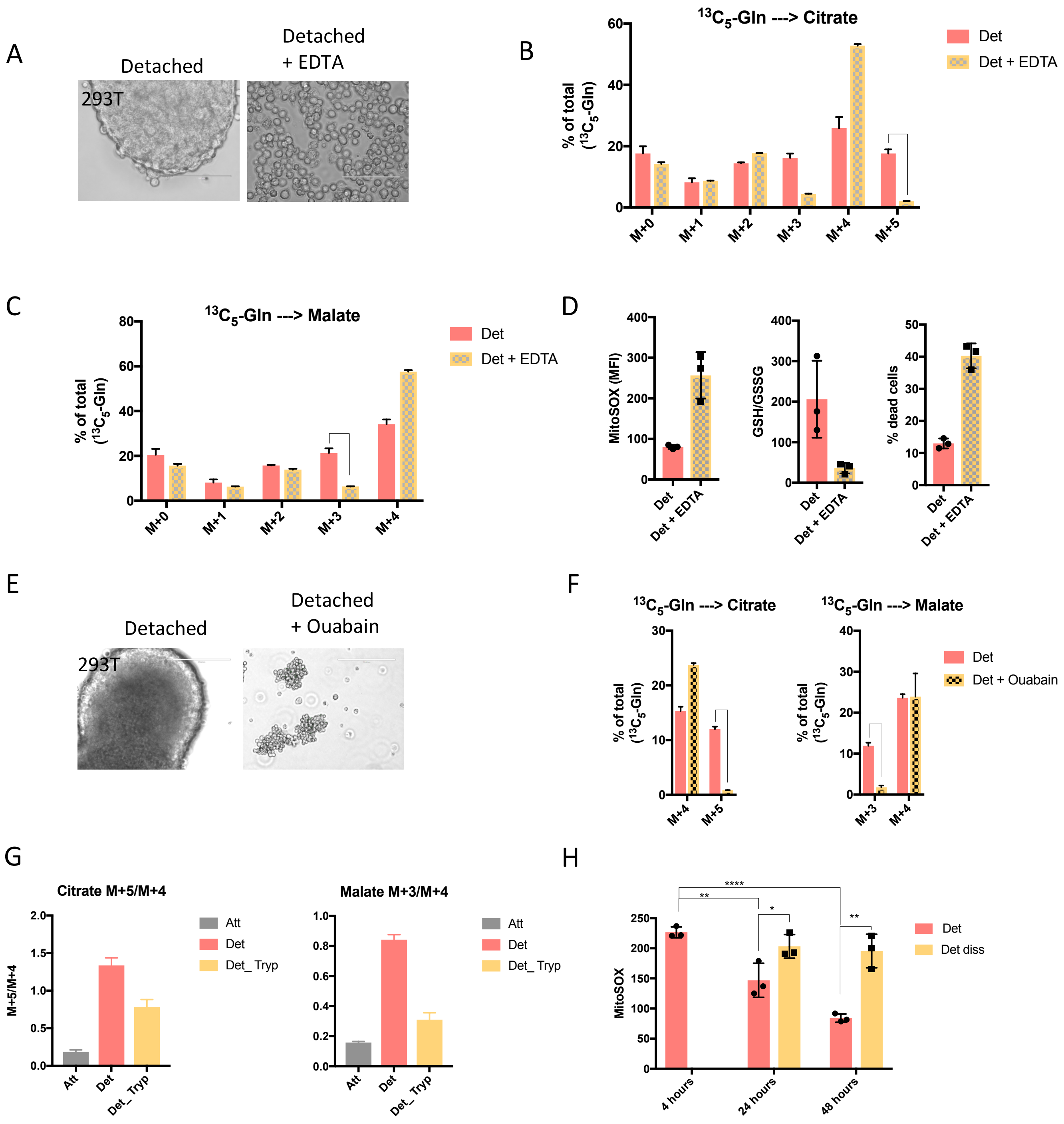

Figure S6. Related to Figure 6

(A) Representative images of 293T cells treated with 2mM EDTA to prevent clustering after matrix detachment. Cells were cultured for 48 hours in detached conditions in the presence of EDTA. Scale bars are 100 μm.

(B and C) Isotopomer distribution of citrate (B) and malate (C) in 293T cells grown in detached conditions with or without EDTA.

(D) Preventing cell clustering after matrix detachment induce an increase in mitochondrial ROS production, oxidative stress and cell death. 293T cells were cultured in detached conditions with or without EDTA for 3 days before GSH/GSSG, mitochondrial ROS and cell viability analysis.
(E) Representative images of 293T cells treated with 0.5 μM Ouabain to prevent clustering after matrix detachment. Cells were cultured for 3 days in detached conditions in the presence of Ouabain. Scale bars are 200 μm.
(F) M+4 and M+5 isotopologues of citrate (left panel) and M+3 and M+4 isotopologues of malate (right panel) originating from $^{13}C_5$-glutamine in 293T cells cultured in detached conditions with and without 0.5 μM Ouabain.
(G) M+5/M+4 isotopologues of citrate (left panel) and M+3/M+4 of malate (right panel) originating from $^{13}C_5$-glutamine in 293T cells cultured in attached conditions, detached conditions and detached conditions before dissociation into single cells (Det diss).
(H) Levels of mitochondrial ROS production in 293T cells cultured in detached conditions and detached conditions before dissociation into single cells for indicated times. Mitochondrial ROS was measured by staining cells with MitoSOX Red before analysis by flow cytometry.
(B,C,D,F,G and H) Data are represented as mean ± SD of triplicate cultures of a representative experiment.
*$p \leq 0.05$, **$p \leq 0.01$, ****$p \leq 0.0001$ Unpaired t test.

Figure S7

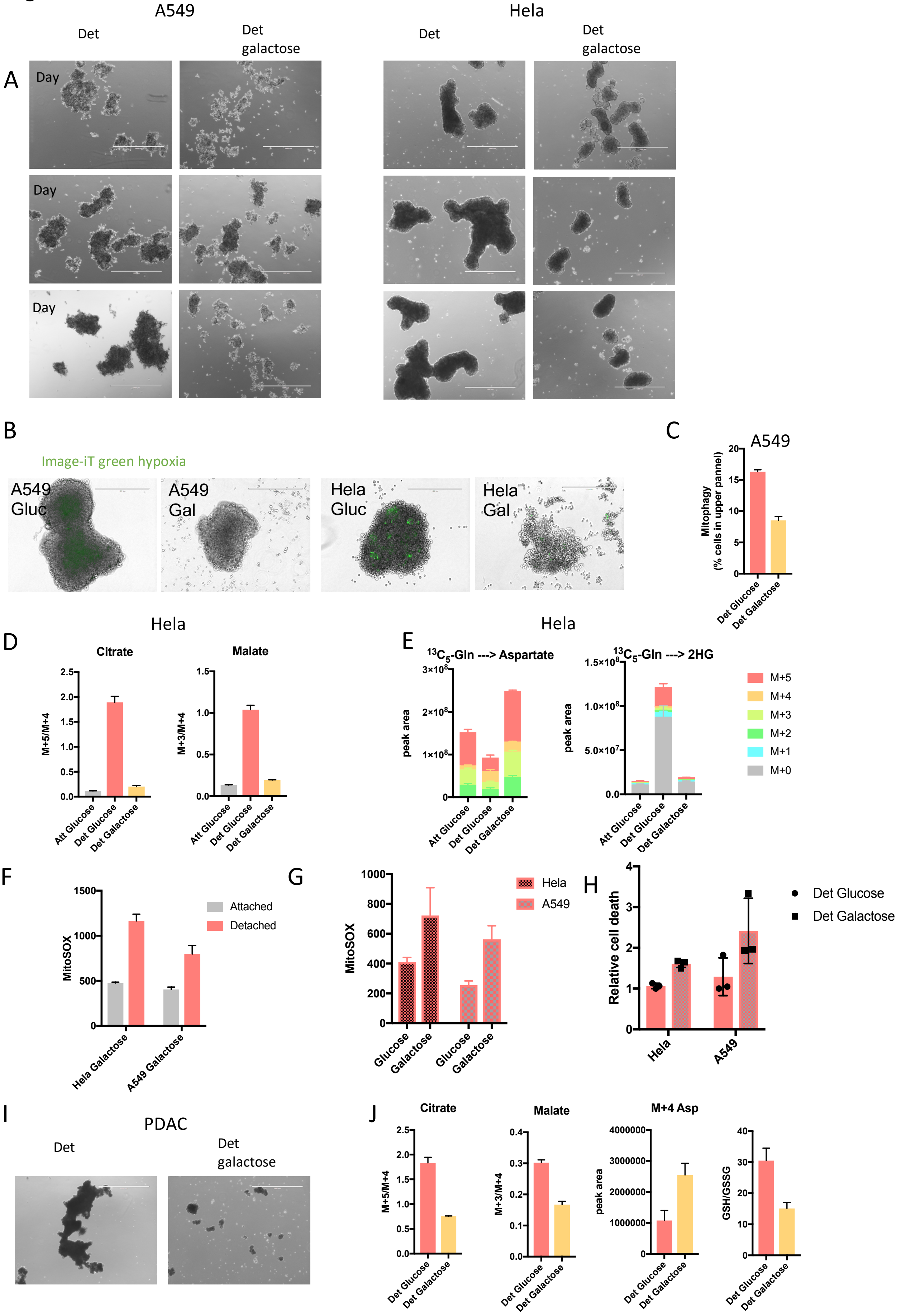
A549
Hela
Det
Det galactose
A
Day
B
Image-iT green hypoxia
A549 Gluc
A549 Gal
Hela Gluc
Hela Gal
C
A549
Mitophagy (% cells in upper pannel)
Det Glucose
Det Galactose
D
Hela
Citrate
Malate
M+5/M+4
M+3/M+4
Att Glucose
Det Glucose
Det Galactose
E
Hela
13C5-Gln ---> Aspartate
13C5-Gln ---> 2HG
peak area
M+5
M+4
M+3
M+2
M+1
M+0
F
MitoSOX
Attached
Detached
Hela Galactose
A549 Galactose
G
Hela
A549
Glucose
Galactose
H
Relative cell death
Det Glucose
Det Galactose
Hela
A549
I
PDAC
Det
Det galactose
J
Citrate
Malate
M+4 Asp
M+5/M+4
M+3/M+4
peak area
GSH/GSSG
Det Glucose
Det Galactose

Figure S7. Related to Figure 7

(A) Representative images of detached A549 and Hela cells grown in glucose or galactose for 3 days. Scale bars are 1000 μm.

(B) Image-iT green hypoxia reagent staining of detached A549 and Hela cells grown in glucose or galactose for 3 days before live cell staining was performed. Scale bars are 400 μm.

(C) Mitochondrial targeted-mkeima expressing Hela cells were cultured in detached conditions in glucose or galactose and analysed by flow cytometry for mitophagy.

(D) $^{13}C_5$-labeled glutamine derived M+5/M+4 citrate and M+3/M+4 malate of Hela cells grown in attached and detached conditions with media containing either glucose or galactose.

(E) Levels and isotopomer distribution of intracellular aspartate and 2-HG of Hela cells cultured in the same conditions as (C). Peak area levels are normalized to cell number.

(F) Mitochondrial ROS analysis using MitoSOX Red dye in attached and detached Hela and A549 cells grown in galactose. Cells were cultured in attached conditions or detached conditions with galactose for indicated times before MitoSOX Red staining and analysis. MitoSOX fluorescence was analysed by flow cytometry.

(G) Mitochondrial ROS analysis using MitoSOX Red dye in detached Hela and A549 cells grown in glucose or galactose.

(H) Fraction of dead Hela and A549 cells grown in detached conditions with either glucose or galactose.

(I) Representative image of PDAC cells grown detached in glucose or galactose. Scale bars are 1000 μm.

(J) $^{13}C_5$-labeled glutamine derived M+5/M+4 citrate, M+3/M+4 malate, M+4 Aspartate and GSH/GSSG of PDAC cells grown in detached conditions with media containing either glucose or galactose.

(C, D, E, F, G, H and J) Data are presented as mean ± SD of triplicate cultures of a representative experiment.

Supplemental Table

Table S1

Description	Step#	Time (min)	Time (sec)	Power (Watts)	SteadyTemp temperature (ºC)	Vacuum cycle vent time (sec)	Vacuum cycle vacuum time (sec)	Vacuum set point (inch Hg)	User Prompt (1 = YES, 0 = NO)	Vacuum OFF (1 = no vacuum, 0 = vacuum)	Vacuum cycle (1 = ON,0=OFF)	Vacuum ON (1 = vacuum, 0 = no vacuum)
BENCH STEP Rinse in 0.1M PB	1	0	0	0	21	0	0	0	1	1	0	0
BENCH STEP Rinse in 0.1M PB	2	0	0	0	21	0	0	0	1	1	0	0
Rinse in 0.1M PB	3	0	40	250	21	0	0	0	1	1	0	0
Rinse in 0.1M PB	4	0	40	250	21	0	0	0	1	1	0	0
Osmium ON	5	2	0	100	21	0	0	20	1	0	0	1
Osmium OFF	6	2	0	0	21	0	0	20	0	0	0	1
Osmium ON	7	2	0	100	21	0	0	20	0	0	0	1
Osmium OFF	8	2	0	0	21	0	0	20	0	0	0	1
Osmium ON	9	2	0	100	21	0	0	20	0	0	0	1
Osmium OFF	10	2	0	0	21	0	0	20	0	0	0	1
Osmium ON	11	2	0	100	21	0	0	20	0	0	0	1
BENCH STEP Rinse in water	12	0	0	0	21	0	0	0	1	1	0	0
BENCH STEP Rinse in water	13	0	0	0	21	0	0	0	1	1	0	0
Rinse in water	14	0	40	250	21	0	0	0	1	1	0	0
Rinse in water	15	0	40	250	21	0	0	0	1	1	0	0
Thiocarbohydrazide ON	16	2	0	100	40	0	0	20	1	0	0	1
Thiocarbohydrazide OFF	17	2	0	0	40	0	0	20	0	0	0	1
Thiocarbohydrazide ON	18	2	0	100	40	0	0	20	0	0	0	1
Thiocarbohydrazide OFF	19	2	0	0	40	0	0	20	0	0	0	1
Thiocarbohydrazide ON	20	2	0	100	40	0	0	20	0	0	0	1
Thiocarbohydrazide OFF	21	2	0	0	40	0	0	20	0	0	0	1
Thiocarbohydrazide ON	22	2	0	100	40	0	0	20	0	0	0	1
BENCH STEP Rinse in water	23	0	0	0	21	0	0	0	1	1	0	0
BENCH STEP Rinse in water	24	0	0	0	21	0	0	0	1	1	0	0
Rinse in water	25	0	40	250	21	0	0	0	1	1	0	0
Rinse in water	26	0	40	250	21	0	0	0	1	1	0	0
Osmium ON	27	2	0	100	21	0	0	20	1	0	0	1
Osmium OFF	28	2	0	0	21	0	0	20	0	0	0	1
Osmium ON	29	2	0	100	21	0	0	20	0	0	0	1
Osmium OFF	30	2	0	0	21	0	0	20	0	0	0	1
Osmium ON	31	2	0	100	21	0	0	20	0	0	0	1
Osmium OFF	32	2	0	0	21	0	0	20	0	0	0	1
Osmium ON	33	2	0	100	21	0	0	20	0	0	0	1
BENCH STEP Rinse in water	34	0	0	0	21	0	0	0	1	1	0	0
BENCH STEP Rinse in water	35	0	0	0	21	0	0	0	1	1	0	0
Rinse in water	36	0	40	250	21	0	0	0	1	1	0	0
Rinse in water	37	0	40	250	21	0	0	0	1	1	0	0
Uranyl acetate ON	38	2	0	100	40	0	0	20	1	0	0	1
Uranyl acetate OFF	39	2	0	0	40	0	0	20	0	0	0	1
Uranyl acetate ON	40	2	0	100	40	0	0	20	0	0	0	1
Uranyl acetate OFF	41	2	0	0	40	0	0	20	0	0	0	1
Uranlyl acetate ON	42	2	0	100	40	0	0	20	0	0	0	1
Uranyl acetate OFF	43	2	0	0	40	0	0	20	0	0	0	1
Uranyl acetate ON	44	2	0	100	40	0	0	20	0	0	0	1
BENCH STEP Rinse in water	45	0	0	0	40	0	0	0	1	1	0	0
BENCH STEP Rinse in water	46	0	0	0	40	0	0	0	1	1	0	0
Rinse in water	47	0	45	250	40	0	0	0	1	1	0	0
Rinse in water	48	0	45	250	40	0	0	0	1	1	0	0
Lead aspartate ON	49	2	0	100	50	0	0	20	1	0	0	1
Lead aspartate OFF	50	2	0	0	50	0	0	20	0	0	0	1
Lead aspartate ON	51	2	0	100	50	0	0	20	0	0	0	1
Lead aspartate OFF	52	2	0	0	50	0	0	20	0	0	0	1
Lead aspartate ON	53	2	0	100	50	0	0	20	0	0	0	1
Lead aspartate OFF	54	2	0	0	50	0	0	20	0	0	0	1
Lead aspartate ON	55	2	0	100	50	0	0	20	0	0	0	1
BENCH STEP Rinse in water	56	0	0	0	21	0	0	0	1	1	0	0
BENCH STEP Rinse in water	57	0	0	0	21	0	0	0	1	1	0	0
Rinse in water	58	0	45	250	21	0	0	0	1	1	0	0
Rinse in water	59	0	45	250	21	0	0	0	1	1	0	0
70% Ethanol ON	60	0	40	250	21	0	0	0	1	1	0	0
70% Ethanol ON	61	0	40	250	21	0	0	0	1	1	0	0
90% Ethanol ON	62	0	40	250	21	0	0	0	1	1	0	0
90% Ethanol ON	63	0	40	250	21	0	0	0	1	1	0	0
100% Ethanol ON	64	0	40	250	21	0	0	0	1	1	0	0
100% Ethanol ON	65	0	40	250	21	0	0	0	1	1	0	0
50% Resin ON	66	3	0	250	21	30	30	20	1	0	1	0
100% Resin ON	67	3	0	250	21	30	30	20	1	0	1	0
100% Resin ON	68	3	0	250	21	30	30	20	1	0	1	0
100% Resin ON	69	3	0	250	21	30	30	20	1	0	1	0
100% Resin ON	70	3	0	250	21	30	30	20	1	0	1	0
TURN SYSTEM OFF	71	0	0	0	21	0	0	0	0	1	0	0

Table S1. Related to STAR Methods. BioWave program details
