# Supplementary material for: Cell Clustering Promotes a Metabolic Switch that Supports Metastatic Colonization
Source: Cell Metab. 2019 Oct 1;30(4):720–734.e5. doi: 10.1016/j.cmet.2019.07.014 (PMC6863392; doi:10.1016/j.cmet.2019.07.014)
Supplement: Document S2. Article plus Supplemental Information [file mmc2.pdf]

Article

Cell Metabolism

Cell Clustering Promotes a Metabolic Switch that Supports Metastatic Colonization

Graphical Abstract

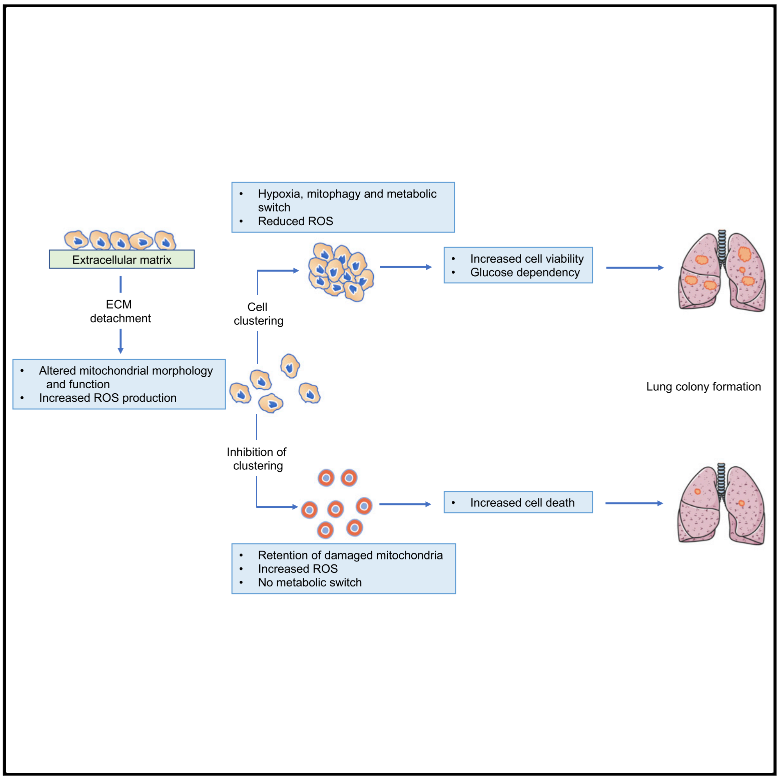

Authors

Christiaan F. Labuschagne, Eric C. Cheung, Julianna Blagih, Marie-Charlotte Domart, Karen H. Vousden

Correspondence

karen.vousden@crick.ac.uk

In Brief

Labuschagne et al. show that the clustering of cancer cells following detachment from the extracellular matrix (ECM) results in hypoxia, which activates mitophagy to remove damaged mitochondria and reductive metabolism to support glycolysis. These responses limit mitochondrial ROS production, allowing cell survival and metastasis.

Highlights

- Loss of attachment causes mitochondrial perturbations and ROS production
- Detached cancer cells cluster, inducing hypoxia, mitophagy, and reductive metabolism
- This response clears damaged mitochondria and reduces mitochondrial ROS production
- The clustering-induced metabolic switch promotes metastatic capacity

Labuschagne et al., 2019, Cell Metabolism *30*, 720–734
October 1, 2019 © 2019 The Author(s). Published by Elsevier Inc.
https://doi.org/10.1016/j.cmet.2019.07.014

CellPress

Cell Metabolism
Article

Cell Clustering Promotes a Metabolic Switch that Supports Metastatic Colonization

Christiaan F. Labuschagne,[1] Eric C. Cheung,[1] Julianna Blagih,[1] Marie-Charlotte Domart,[1] and Karen H. Vousden[1,2,*]
[1]The Francis Crick Institute, 1 Midland Road, London NW1 1AT, UK
[2]Lead Contact
*Correspondence: karen.vousden@crick.ac.uk
https://doi.org/10.1016/j.cmet.2019.07.014

SUMMARY

Cancer metastasis depends on cell survival following loss of extracellular matrix attachment and dissemination through the circulation. The metastatic spread can be enhanced by the clustering of detached cancer cells and increased antioxidant defense. Here, we link these responses by describing how cell clustering limits reactive oxygen species (ROS). Loss of attachment causes mitochondrial perturbations and increased ROS production. The formation of cell clusters induces a hypoxic environment that drives hypoxia-inducible factor 1-alpha (Hif1α)-mediated mitophagy, clearing damaged mitochondria and limiting ROS. However, hypoxia and reduced mitochondrial capacity promote dependence on glycolysis for ATP production that is supported by cytosolic reductive metabolism. Preventing this metabolic adaptation or disruption of cell clusters results in ROS accumulation, cell death, and a reduction of metastatic capacity *in vivo*. Our results provide a mechanistic explanation for the role of cell clustering in supporting survival during extracellular matrix detachment and metastatic spread and may point to targetable vulnerabilities.

INTRODUCTION

The metastatic spread of tumors to distant sites is responsible for most cancer-related deaths (Eccles and Welch, 2007). While this invasive process remains poorly understood, it is clear that the metastasizing cell must overcome numerous hurdles associated with movement out of the primary tumor, entry into the circulation, and re-establishment within a distant organ (Fidler, 2003; Hunter et al., 2008). These barriers make metastasis a highly inefficient process. One key limitation to successful metastasis is the caspase-dependent and caspase-independent death of cells that lose stromal interactions as they become detached from the extracellular matrix (ECM) (Simpson et al., 2008). Clearly, cells that successfully metastasize have acquired mechanisms to survive loss of attachment, and dependence on these changes may provide effective therapeutic targets (Buchheit et al., 2014). One factor that can enhance the metastatic capacity of cancers is the ability of detached cells to form clusters or aggregates (Aceto et al., 2014; Gkountela et al., 2019), although the underlying mechanism by which aggregation supports metastatic capacity is not well understood.

Both caspase-dependent and caspase-independent detachment-associated cell death have been linked to increased reactive oxygen species (ROS) (Hawk and Schafer, 2018; Hawk et al., 2018). ROS-induced death in detached mammary cells was shown to result from a loss of glucose uptake and decreased flux through the oxidative pentose phosphate pathway (Schafer et al., 2009). Activation of oncogenes such as ErbB2 rescued this death by promoting glycolysis. The increased ROS in these cells also inhibited fatty acid oxidation (FAO), thus limiting the use of these alternative pathways for energy production in the face of decreased glycolysis. Recently, the ability to survive detachment was shown to reflect the reductive carboxylation of glutamine, producing citrate to support the IDH2-mediated production of NADPH in mitochondria and limiting mitochondrial ROS (Jiang et al., 2016). Ferroptosis—a form of cell death associated with lipid peroxidation—has been shown to be induced in response to detachment (Brown et al., 2017). This response was limited by cell clustering, which activated a PVRL4-α6β4-Src signaling pathway to induce the antioxidant enzyme GPX4 (Brown et al., 2018). Loss of this protective response though depletion of

Context and Significance

The ability of cancer cells to spread from the initial tumor site to other organs accounts for its deadliness. Normally, cells die once they detach from their normal environment due to changes in their mitochondria and being exposed to oxidative stress (reactive oxygen species, ROS). Researchers at the Crick Institute in England now show that clustering of detached cancer cells leads to an active shift in their cellular metabolism, which allow them to clear damaged mitochondria and limit oxidative stress. Preventing this metabolic adaptation or disrupting the cell clusters results in ROS accumulation, cell death, and a reduction of metastatic capacity. The work furthers our understanding of the role of cell clustering in metastatic spread and highlights potential targetable metastatic vulnerabilities.

 © 2019 The Author(s). Published by Elsevier Inc.
This is an open access article under the CC BY license (http://creativecommons.org/licenses/by/4.0/).

$\alpha6\beta4$ led to ferroptosis in detached cells. The importance of mitochondrial ROS control during ECM detachment was further reflected in a study showing that RIPK1-mediated mitophagy resulted in lower mitochondrial NADPH production and reduced viability (Hawk et al., 2018). However, mitophagy can also function to remove damaged or dysfunctional mitochondria that produce high levels of ROS (Kubli and Gustafsson, 2012). Under these conditions, the elimination of mitophagy would be expected to enhance, rather than lower, oxidative stress. As seen in tissue culture systems, the role of mitochondrial ROS control in metastasis *in vivo* is complex. Some studies have shown that antioxidants can inhibit metastasis, suggesting that ROS may contribute to cancer spread (Goh et al., 2011; Porporato et al., 2014). However, other models show that the ability to limit ROS is necessary for effective metastasis. Several mouse melanoma models have demonstrated increased metastasis in animals treated with antioxidants and a dependence on mitochondrial NADPH-producing enzymes for effective tumor dissemination (Le Gal et al., 2015; Piskounova et al., 2015). These observations are consistent with the requirement for protection from ROS-induced cell death. In this study, we show that increased ROS in detached cells reflects the accumulation of damaged mitochondria. We find that cell clustering limits ROS by driving hypoxia and hypoxia-inducible factor 1-alpha (Hif1α)-mediated mitophagy, thus removing damaged ROS-producing mitochondria. The resultant decrease in mitochondrial capacity results in a dependence on glycolysis that is supported by reductive carboxylation of glutamine to malate. Cells that are prevented from clustering or forced to use OXPHOS are unable to make these adaptations, leading to the accumulation of excessive levels of ROS, decreased survival, and a reduction in metastatic capacity.

RESULTS

Loss of Attachment Induces Reductive Carboxylation into Malate and 2HG Production

To assess metabolic changes that may contribute to cancer cell survival during anchorage independent growth, we compared cells grown in monolayers (attached) to cells grown on ultra-low attachment plates that prevent cell adhesion and force cells to grow in suspension (detached). Using this system, we cultured the tumor cell lines 293T, HeLa, and A549 in attached and detached conditions in the presence of $^{13}C_5$-glutamine and traced the incorporation of carbons into TCA cycle intermediates with LC-MS. In agreement with a recent study (Jiang et al., 2016), we identified a switch to reductive carboxylation in detached cells, as indicated by an increase in M + 5 citrate from $^{13}C_5$-glutamine (Figures 1A and S1A). While this cytosolic citrate can shuttle to the mitochondria to support mitochondrial NADPH production (Jiang et al., 2016), our further analysis of TCA cycle intermediates also revealed an increase in M + 3 and a decrease in M + 4 malate and fumarate in detached cells (Figures 1B and S1B). These results indicated that a fraction of citrate originating from reductive carboxylation is cleaved and further reduced to malate in these cells, a reaction that is catalyzed by malate dehydrogenase (MDH) in an NADH-dependent reaction. Additionally, we observed a dramatic increase in glutamine derived 2-hydroxyglutarate (2HG) in detached cells (Figures 1C and S1C). 2HG is a chiral molecule and exists as the two enantiomers, D- and L-2HG. D-2HG is an oncometabolite generated by oncogenic IDH mutants and has been implicated in many tumorigenic processes, while L-2HG is considered to be a normal metabolic byproduct. To determine which isoform of 2HG is produced in detached cells, we performed chiral derivatization of 2HG enabling us to chromatographically separate and measure D- and L-2HG using GC-MS. This revealed that the majority of 2HG produced in detached cells is the L-enantiomer (Figure 1D). Previous studies have shown that L-2HG can be produced from the reduction of glutamine derived αKG catalyzed by promiscuous substrate usage by LDHA and MDH in an NADH-dependent reaction (Intlekofer et al., 2015, 2017). To determine if this is the case in detached cells, we decreased MDH1,2 and LDHA levels respectively using small interfering RNA (siRNA) and measured 2HG production. Indeed, reduction of both MDH1,2 and LDHA led to a significant decrease in 2HG production (Figure 1E), indicating that the promiscuous side reactions of these enzymes are the source of 2HG in detached cells. Together, these data demonstrate a switch in glutamine usage in detached cells toward reductive metabolism to generate 2HG and malate. Importantly, these reactions also generate NAD^+ (Figure 1F).

Reductive Carboxylation into Malate and 2HG Supports Glycolysis in Detached Cells

We next sought to determine the functional relevance of reductive malate and 2HG production in detached cells. Glycolysis requires NAD^+ as an electron acceptor and the recycling of NADH back into NAD^+ is pivotal for maintaining a favorable NAD^+/NADH ratio for optimum glycolytic flux. One of the hallmarks of enhanced glycolysis is the increased conversion of pyruvate into lactate via lactate dehydrogenase, a reaction that recycles NADH into NAD^+ to sustain glycolysis. Additionally, reductive malate and 2HG production via MDH also produces NAD^+ from NADH (Figure 1F), and recent studies have shown that this pathway of NADH shuttling supports glycolysis in highly proliferative cells (Hanse et al., 2017) and cells with mitochondrial dysfunction (Gaude et al., 2018). To determine the glycolytic status of detached cells, we assessed glucose metabolism by tracing $^{13}C_6$-glucose carbons into glycolysis and TCA cycle intermediates. This showed increased labeling of pyruvate and lactate, and decreased labeling of citrate from $^{13}C_6$-glucose in detached cells (Figures 2A and S2A), suggesting that detached cells have a more glycolytic phenotype. Consistent with this observation, detached cells showed increased levels of phosphorylated pyruvate dehydrogenase (pPDH) (Figures 2B and S2B), which inhibits the flux of pyruvate into the TCA cycle (Jiang et al., 2016). To determine whether cytosolic reductive malate production supports glycolysis in detached cells, we lowered MDH1 levels using siRNA (Figure S2C). $^{13}C_5$-Glutamine tracing revealed a decrease in the ratio of M + 3/M + 4 malate following MDH1 knockdown (Figure 2C), confirming the activation of this pathway in detached cells. Importantly, MDH1 depletion also resulted in decreased lactate production (Figure 2D), to the same extent as LDHA knockdown, demonstrating that MDH1 supports glycolysis in detached cells. Interestingly, LDHA depletion resulted not only in a reduced lactate production but also in a drop of M + 3 malate production (Figure 2C), suggesting an

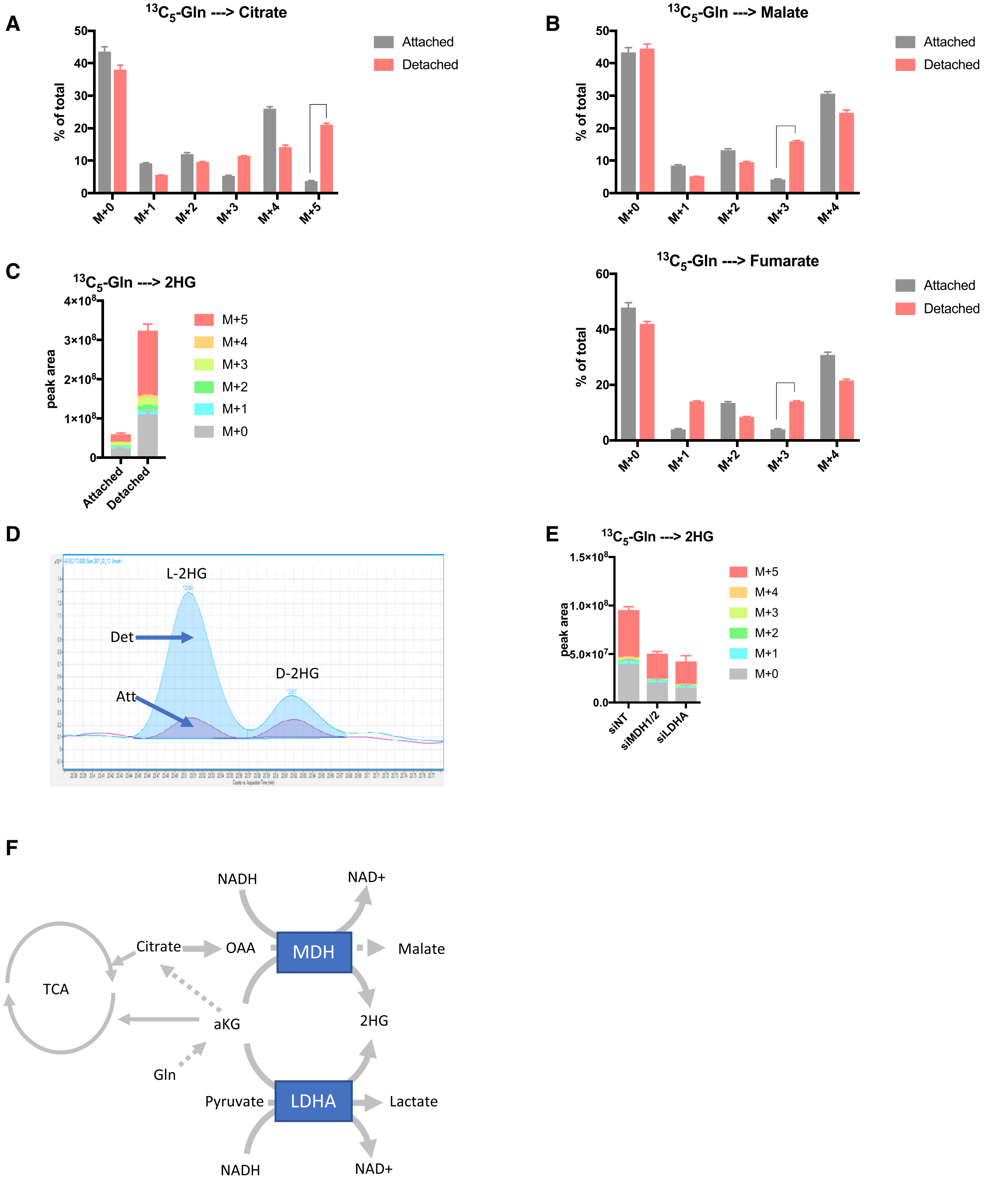

Figure 1. Cells Growing in Detached Conditions Have Increased Reductive Glutamine Metabolism

(A and B) Isotopomer distribution of (A) citrate and (B) malate and fumarate in attached and detached 293T cells cultured with $^{13}C_5$-glutamine for 4 h.

(C) Levels and isotopomer distribution of 2-hydroxyglutarate in attached and detached cells cultured in the same conditions as (A). Peak area levels are normalized to cell number.

(D) Representative GC-MS chromatogram showing the levels of L-and D-2-hydroxyglutarate in 293T cells cultured in detached and attached conditions.

(E) 2-HG levels in 293T detached cells after siRNA knockdown of MDH1/2 and LDHA. Peak area levels are normalized to cell number.

(F) Schematic representation of reductive glutamine metabolism in detached cells generating malate, 2-HG, and NAD^+.

(A, B, C, and E) Data are presented as ± SD of triplicate wells of representative experiments.

interdependence between glycolysis and the reductive MDH1 reaction to create an NAD^+/NADH bi-cycle (Figure 2H) in detached cells. This relationship was further demonstrated by removing glucose from the media, which decreased NADH derived from glycolysis and resulted in a complete inhibition of reductive carboxylation into malate (Figure S2D).

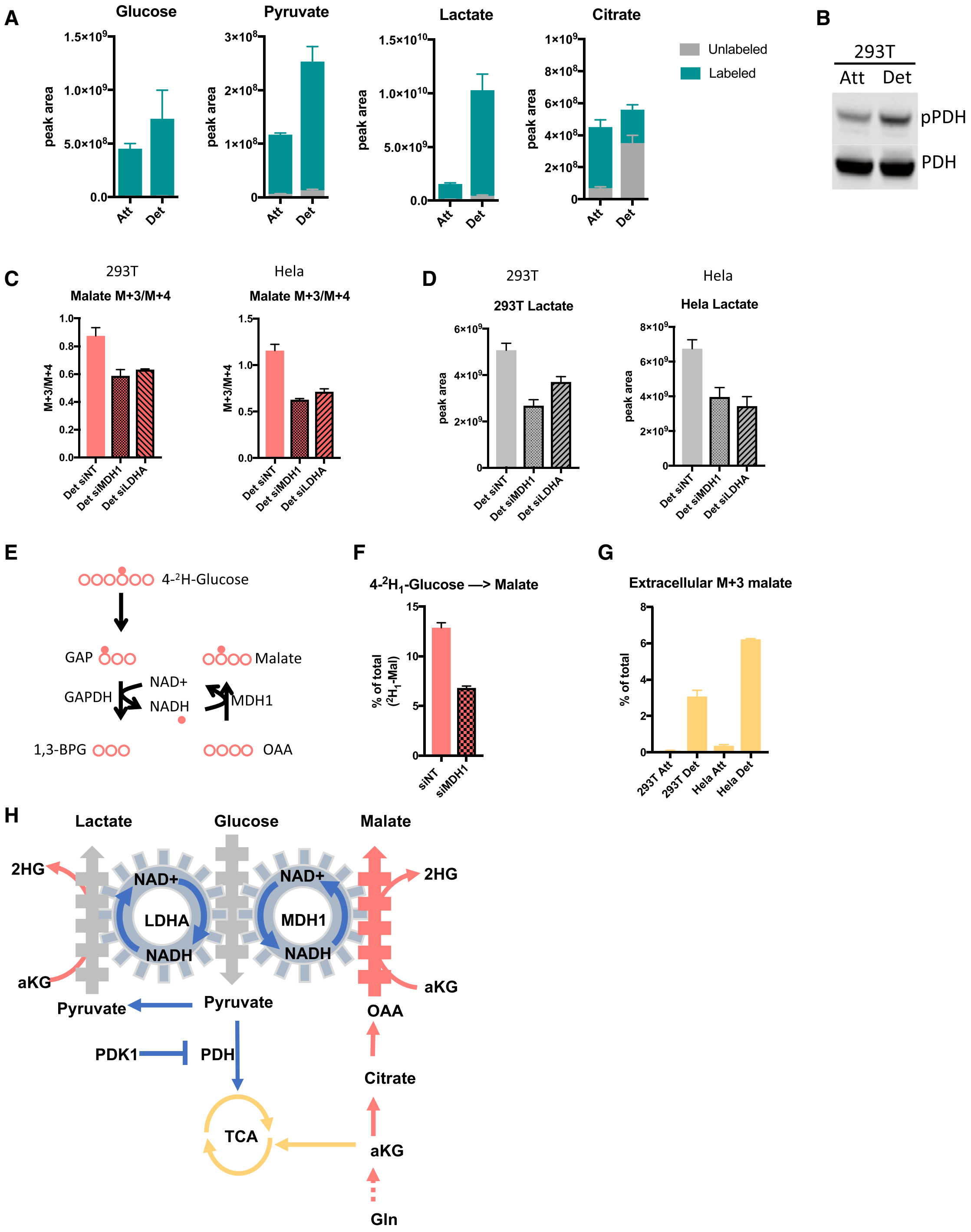

Figure 2. Reductive Carboxylation into Malate Supports Glycolysis in Detached Cells

(A) Levels of isotope labeling of glycolytic intermediates of 293T cells cultured in attached or detached conditions in the presence of $^{13}C_6$-glucose for 4 h. Peak area levels are normalized to cell number.

(B) Western blot analysis demonstrating pyruvate dehydrogenase phosphorylation in 293T attached and detached cells.

(C) The M + 3/M + 4 ratio of malate in 293T and HeLa detached cells after siRNA knockdown of MDH1 and LDHA.

(D) Lactate levels of 293T and HeLa detached cells after siRNA knockdown of MDH1 and LDHA.

(E) A schematic representation of hydrogen-labeling experiment.

(F) Levels of deuterium-labeled malate from 4-$^2H_1$-glucose labeling in detached 293T cells following siRNA knockdown of MDH1.

(G) Levels of M + 3 malate excreted from attached and detached 293T and HeLa cells.

(H) A schematic representation of reductive carboxylation into malate supporting glycolysis by regenerating NAD^+ in detached cells.

(A, C, D, F, and G) Data are presented as mean ± SD of triplicate cultures of representative experiments.

To further investigate the link between glycolytic-derived NADH and MDH1 in detached cells, we performed a hydrogen tracing experiment with 4-^{2}H-glucose, which enabled us to trace hydrogen transfer from GAPDH-derived NADH onto malate via MDH1 (Figure 2E) (Lewis et al., 2014). We found that M + 1 malate decreased significantly upon MDH1 depletion in detached cells, further supporting the role of this pathway in NAD^+ recycling (Figures 2F and S2E).

While the isotopomer distribution of malate was clearly altered in detached cells treated with $^{13}C_5$-glutamine, there was no change in total malate levels (Figure S2F). This observation suggests that malate is removed to prevent product inhibition. Cytosolic malate can be converted into pyruvate via malic enzyme, transported into the mitochondria or excreted by the cell (Figure S2G). We failed to observe any labeling from glutamine into pyruvate (Figure S2H), while a large amount of M + 3 malate was excreted by detached cells (Figure 2G). Taken together, these data show that detached cells undergo a metabolic switch toward glycolysis, which is supported by cytosolic NAD^+ recycling through LDHA and MDH1.

Detached Cells Have Altered Mitochondrial Morphology and Function and Are Sensitive to Glucose Starvation

Reductive carboxylation has been shown to support glycolysis in cells with mitochondrial dysfunction (Gaude et al., 2018; Mullen et al., 2011). We therefore considered whether our observations of increased glycolysis and reductive carboxylation in detached cells might reflect an impact of loss of attachment on mitochondrial function. Transmission electron microscopy revealed a striking alteration in mitochondrial morphology, with significantly fewer and shorter cristae per mitochondrion in detached cells (Figure 3A). To account for differences in cell shape during detachment, we trypsinized cells grown in attached and detached conditions into single cells followed by fixation for imaging. While the difference in cristae length and cristae/outer mitochondrial membrane (OM) persisted, differences in the number of cristae per mitochondrion were no longer detected (Figure S3A), suggesting that the drop in cristae number is a direct response to change in cell shape due to trypsin treatment. To assess mitochondrial function, we measured intracellular levels of aspartate and its biosynthesis—a good indicator of electron transport function (Birsoy et al., 2015; Sullivan et al., 2015). We found a sharp drop in intracellular aspartate and aspartate biosynthesis from labeled glutamine (M + 4 Asp) (Figures 3B and S3B), which coincided with reduced oxygen consumption rates in detached cells (Figures 3C and S3C). Furthermore, detached cells had increased mitochondrial ROS production (Figure 3D), consistent with previous reports of increased ROS in detached cells (Jiang et al., 2016; Schafer et al., 2009).

Our observations that detached cells are glycolytic, with inefficient respiration, suggested that detached cells may be sensitive to glucose starvation. To test this, we cultured detached cells in glucose for 7 days to allow for metabolic adaptation before switching them to a glucose-free medium for 24 h. These cells showed a significant loss of viability compared to attached cells grown in the same media conditions (Figures 4A and S4A). While intracellular ATP levels were significantly reduced after 4 h in glucose-starved attached cells and in detached cells in complete media, they were almost undetectable in detached cells grown without glucose (Figure 4B). An accompanying strong increase in AMP levels (Figure 4B) pointed to an energy crisis in these cells, which was rescued by supplementing the glucose-free media with increasing amounts of ATP—an intervention that increased cell viability in a concentration-dependent manner (Figures 4C and S4B). Analysis of intracellular nucleotide levels after ATP supplementation revealed that ATP is taken up by detached cells and completely dephosphorylated and degraded to form hypoxanthine (Figure S4C), implying that under these conditions, cells are unable to recycle ATP. This is consistent with our observation that detached cells have reduced OXPHOS and suggests that these cells are unable to use alternative energy sources like FAO. To test this, we cultured cells with $^{13}C_{16}$-palmitate in the presence or absence of glucose for 4 h and measured the incorporation of carbons from palmitate into TCA cycle intermediates. Attached cells significantly increased the contribution of carbons from palmitate into citrate and other TCA cycle intermediates in response to glucose starvation. By contrast, detached cells failed to utilize lipids in the absence of glucose (Figures 4D, 4E, S4D, and S4E). Furthermore, detached cells showed an accumulation of lipid droplets consistent with defects in both FAO and OXPHOS (Figure 4F). Collectively, these data show that upon loss of attachment, cells undergo a morphological change in their mitochondria, which coincide with a decrease in mitochondrial OXPHOS efficiency. These changes lead to an increased dependence on glucose for ATP production, and the cells are consequently vulnerable to glucose starvation.

Cell Clustering upon Detachment Induces Hypoxia and Hif1α-Mediated Mitophagy

To understand the mechanism by which detached cells are able to induce the metabolic switch from OXPHOS toward glycolysis, we considered the observation that loss of attachment promotes cell clustering (Aceto et al., 2014; Brown et al., 2018) (Figure 5A), a process mediated by cell-cell adhesion proteins located on the cell surface (Liu et al., 2019). We found that loss of attachment led to a dramatic upregulation of total cadherin expression (Figure 5B), a class of adhesion proteins that form homodimers with cadherins on adjacent cells in a calcium-dependent manner, promoting cell clustering (Maître and Heisenberg, 2013). Multicellular tumor spheroids are often associated with hypoxia, and using a probe that fluoresces in the absence of oxygen, we showed a clear increase in hypoxia in the center of the cell clusters but not in the outer cell layers (Figures 5C and S5A). To ensure that hypoxia is due to cell clustering and not local depletion of the oxygen concentration in the media (Place et al., 2017), we cultured the cells on a shaker when staining with the hypoxia marker. This resulted in equal staining compared to static cultures (Figures 5C and S5A), indicating that hypoxia is not due to oxygen depletion from the media. Hypoxia would be expected to stabilize and activate Hif1α, and although we could only see a very modest increase in Hif1α protein levels in detached cells (as reported previously [Jiang et al., 2016]), we detected a significant increase in the expression of BNIP3 and NIX (BNIP3L), products of Hif1α target genes (Figure 5D) (Sowter et al., 2001). To confirm that BNIP3 and NIX are indeed targets of Hif1α in our cells, we pharmacologically stabilized Hif1α in the presence or absence of Hif1α siRNA.

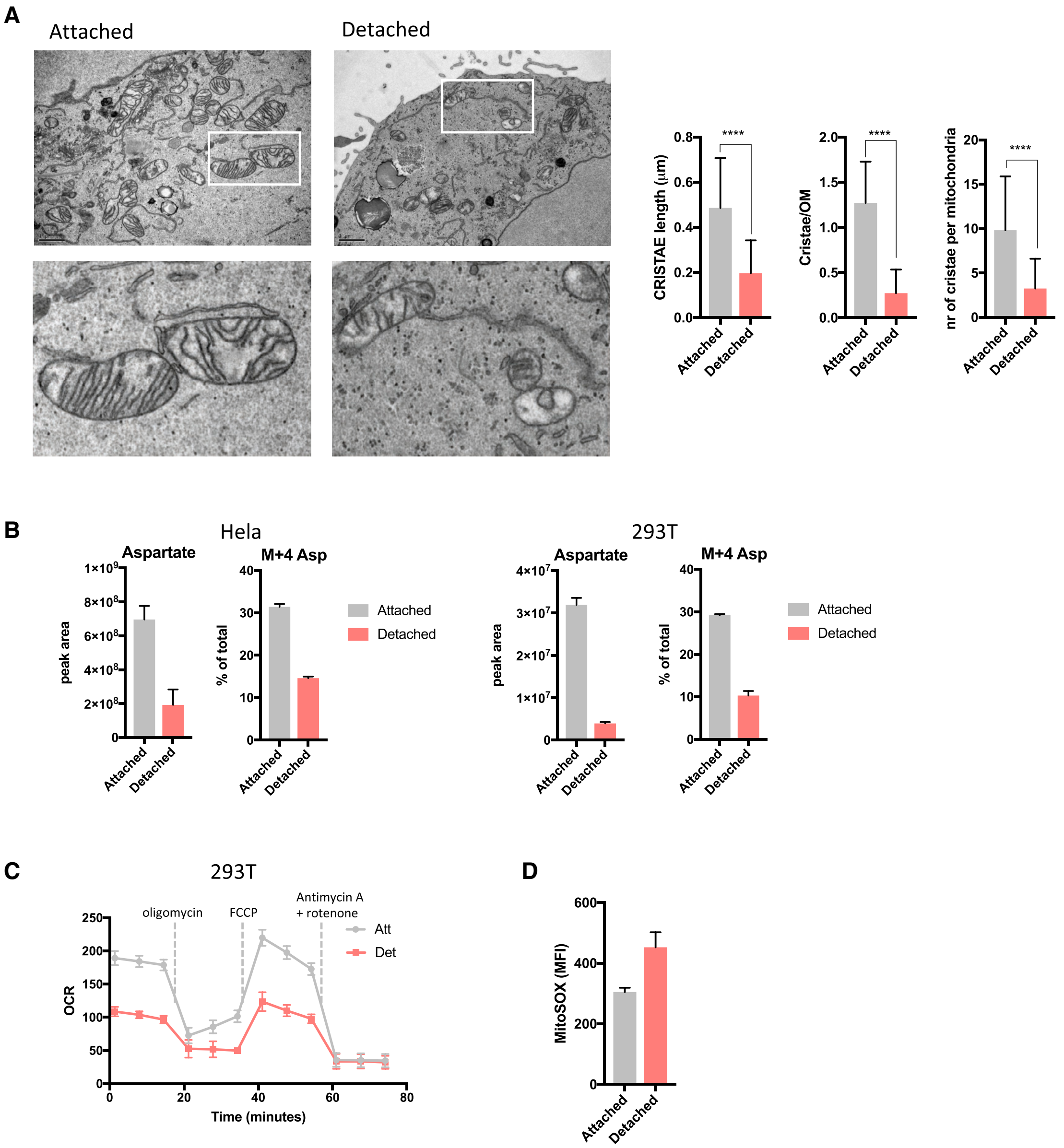

Figure 3. Detached Cells Have Altered Mitochondrial Morphology and Function

(A) Representative transmission electron microscopy images of mitochondria of attached and detached cells. Scale bars represent 1 μm. Graphs present quantified cristae length, cristae/outer membrane ratio and number of cristae per mitochondria (Att n = 58 mitochondria and Det n = 283 mitochondria). Data are presented as mean ± SD. ****p $\leq$ 0.0001, unpaired t test.

(B) Intracellular levels of unlabeled and M + 4-labeled aspartate from $^{13}C_5$-labeled glutamine in attached and detached HeLa and 293T cells. Peak area levels are normalized to cell number.

(C) Oxygen consumption rate profiles of 293T cells cultured in attached or detached conditions. Dotted lines show incubation of cells in the presence of indicated mitochondrial inhibitors. Data are normalized to protein content.

(D) Mitochondrial ROS production in attached and detached 293T cells as measured by MitoSOX Red staining and analysis by flow cytometry.

(B–D) Data are presented as mean ± SD of triplicate cultures of representative experiments.

Hif1α stabilization strongly induced BNIP3 and NIX, a response that was ablated following Hif1α depletion by siRNA (Figure 5E). Furthermore, the increase in expression of BNIP3 and NIX in detached cells was dependent on Hif1α expression (Figure 5F). BNIP3 and NIX are transmembrane proteins located on the OM that have been implicated in the induction of mitophagy (Zhang et al., 2008). To determine whether detached cells show increased mitophagy, we transfected HeLa cells with mitochondrial-targeted mKeima, a fluorescence biosensor that emits different colored signals at acidic and neutral pHs and so can be used to detect lysosomal mitochondrial degradation as a marker of mitophagy (Lazarou et al., 2015). Using this probe, we found that loss of attachment increased the percentage of cells with mitochondria targeted to the lysosome (as indicated by a shift

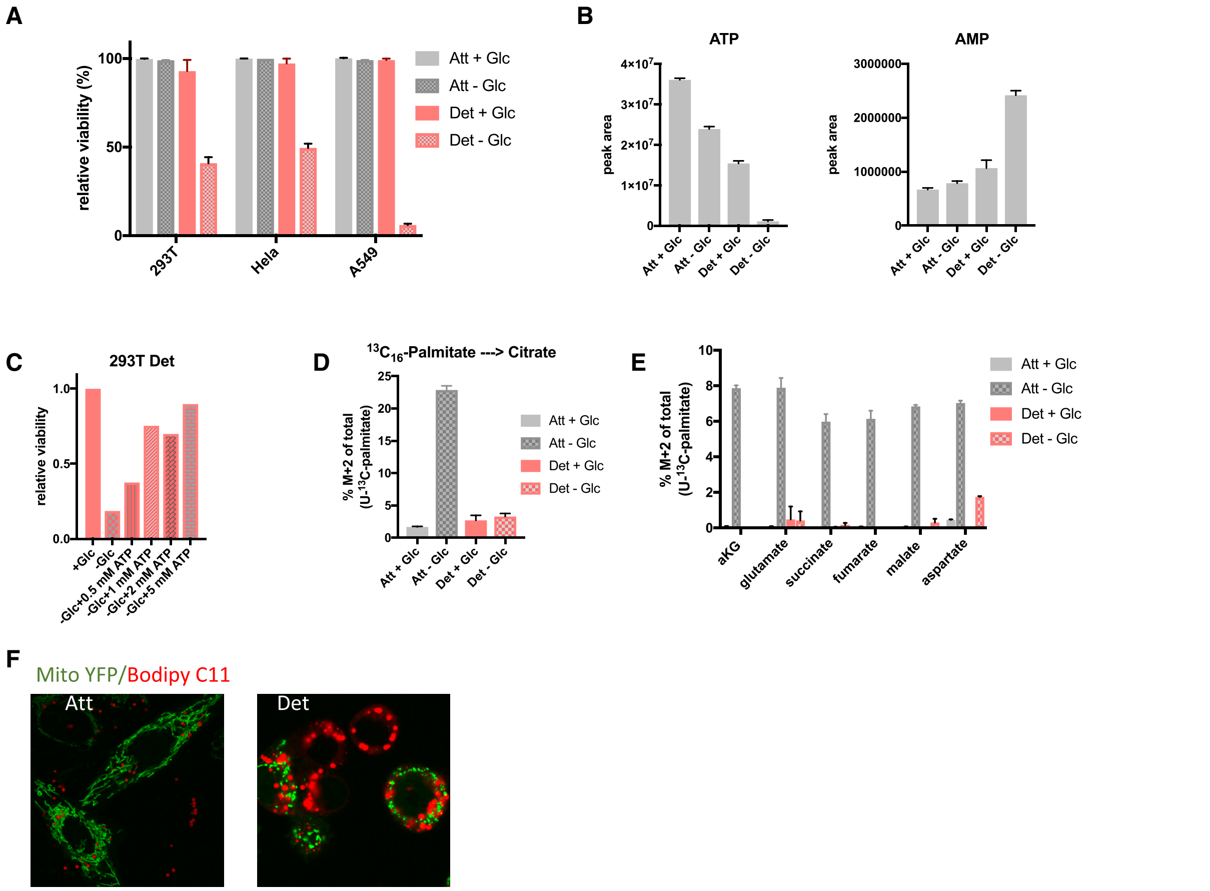

Figure 4. Detached Cells Are Dependent on Glycolysis for ATP Production and Survival

(A) Cell viability of 293T, HeLa, and A549 cells cultured in attached and detached conditions in the presence or absence of glucose. Cells were cultured in attached or detached conditions for 7 days in the presence of glucose before glucose starvation for 24 h followed by staining with eFlour 780 viability dye and analysis by flow cytometry.

(B) Intracellular ATP and AMP levels of 293T attached and detached cells in the presence or absence of glucose. Cells were grown in attached and detached conditions like in (A) before glucose was removed for 4 h followed by LC-MS analysis.

(C) Cell viability of 293T cells as measured in (A) in cells cultured with glucose or without glucose plus indicated ATP concentrations.

(D and E) Levels of M + 2 citrate (D) and other TCA cycle intermediates (E) originating from $^{13}C_{16}$-palmitate in 293T attached and detached cells cultured with or without glucose. Cells were cultured as in (A) before addition of labeled palmitate and removal of glucose for 4 h.

(F) Confocal microscopy images of attached and detached HeLa cells expressing Mito YFP and stained with Bodipy C11.

(A, B, D, and E) Data are represented as mean ± SD of triplicate cultures of representative experiments.

in the excitation wavelength to 561 nm), indicating an increase in mitophagy (Figures 5G and S5B). The increased mitophagy was accompanied by a decrease in mitochondrial mass in detached cells, as measured by Mito Tracker green and immunoblotting for Tom20 across a number of different cell lines (Figures 5H and 5I). This reduction in mitochondria could also be visualized by fluorescence microscopy (Figure S5C). Importantly, siRNA depletion of Hif1α or BNIP3 plus NIX each decreased mitophagy in detached cells (Figures 5J and S5D), resulting in a significant increase in mitochondrial mass and ROS production (Figures 5K and 5L). These results are consistent with a recent report showing mutant Kras induction of NIX expression in pancreas cancer cells helps to limit mitochondrial mass and ROS (Humpton et al., 2019). Our data show that cell clustering following loss of attachment induces hypoxia-mediated mitophagy, which is essential for clearing damaged mitochondria and limiting ROS. In line with these findings, we have found that high BNIP3 expression in some cancer types is associated with reduced survival in humans (Figure S5E) (Li et al., 2017). It has also been shown, however, that mitophagy disfunction due to loss of BNIP3 can promote cancer progression (Chourasia et al., 2015). This likely reflects differential roles of mitophagy in different tissues and stages of tumor progression.

The Cell-Clustering-Induced Metabolic Switch Is Important for ROS Limitation and Cell Survival

Previous studies have shown that hypoxia induces a metabolic switch that is associated with reductive carboxylation, increased glycolysis, and a drop in FAO (Eales et al., 2016; Huang et al., 2014), consistent with our observations in detached cells. To determine whether clustering-induced hypoxia is responsible for the metabolic switch in detached cells, we prevented cell

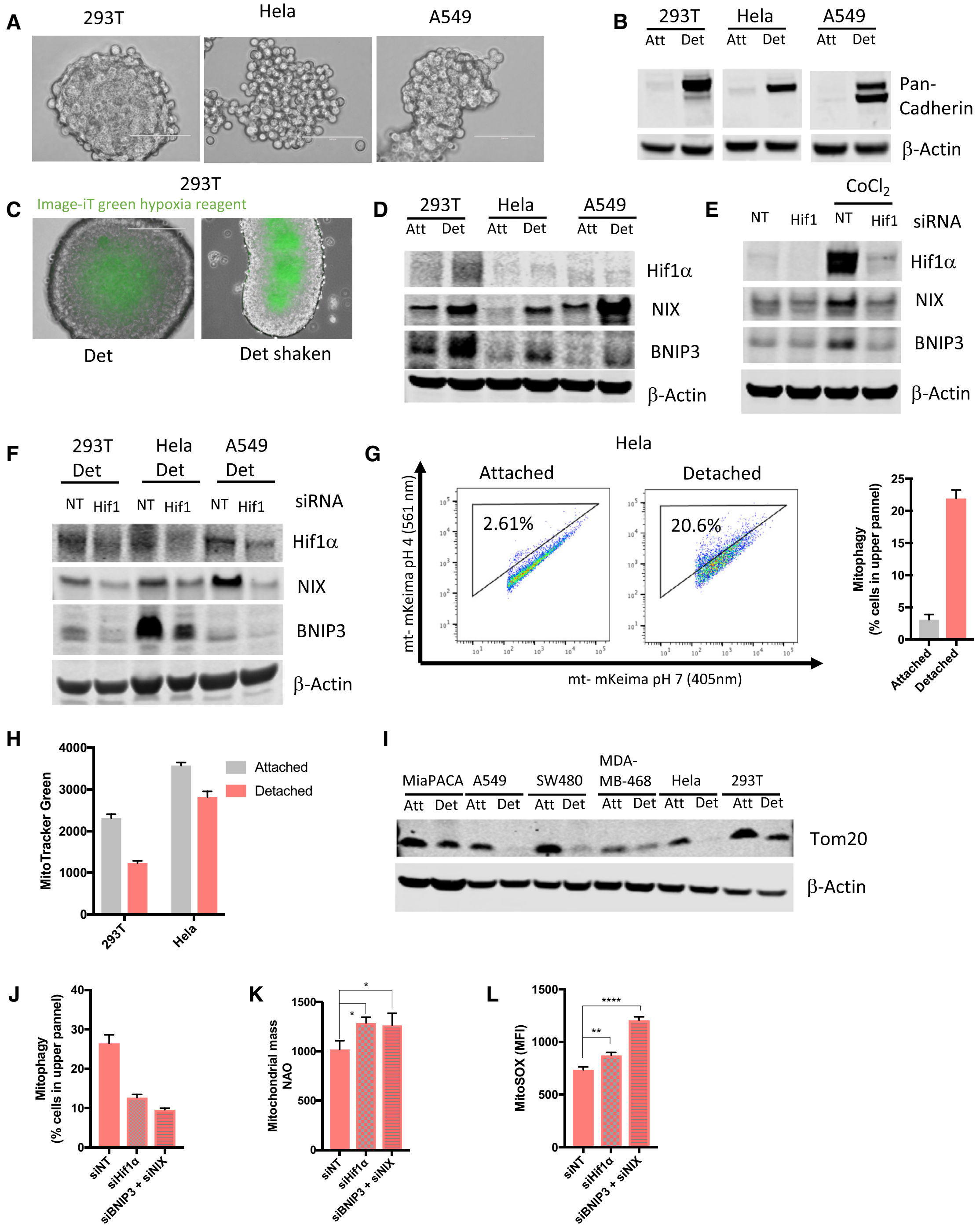

Figure 5. Matrix Detachment Leads to Cluster Formation, Hypoxia Induction, and Hif1α-Mediated Mitophagy

(A) Representative microscopy images of detached 293T, HeLa, and A549 cells forming clusters. Scale bars represent 100 μm.

(B) Western blot analysis of pan-cadherin and β-actin levels of attached and detached 293T, HeLa, and A549 cells. Equal protein levels were loaded per condition.

(C) Image-iT green hypoxia reagent staining of detached 293T cells cultured in static (left panel) and shaken (right panel) conditions. Cells were cultured in detached conditions for 3 days before live cell staining was performed.

(D) Western blot analysis of attached and detached 293T, HeLa, and A549 cells showing expression of HIF1α, NIX, BNIP3, and β-actin (NIX was detected on a duplicate blot). Equal protein amounts were loaded per condition and verified by ponceau staining. β-Actin was used to verify the integrity of the samples.

(E) Western blot showing expression of HIF1α, NIX, BNIP3, and β-actin (NIX was detected on a duplicate blot) in 293T attached cells treated with $CoCl_2$ in the presence or absence of siHif1α. Equal protein amounts were loaded per condition and verified by ponceau staining. β-Actin was used to verify the integrity of the samples.

(legend continued on next page)

clustering by culturing cells in the presence of EDTA. EDTA chelates Mg2+ and Ca2+, which are important for cadherin-dependent cell-cell adhesion, and therefore prevents cell clustering (Figures 6A and S6A) (Brown et al., 2018). Detached cells grown in the presence of EDTA failed to make the metabolic switch toward reductive carboxylation seen in the clustered detached cells (Figures 6B, 6C, S6B, and S6C). Furthermore, after 48 h, detached cells that had been prevented from clustering showed a strong increase in mitochondrial ROS and a clear increase in oxidative stress, as measured by a decreased GSH/GSSG ratio (Figures 6D and S6D), compared to clustered cells. The increased ROS was accompanied by a dramatic increase in cell death in detached cells that were prevented from clustering (Figures 6D and S6D). While these results support a role for clustering in the metabolic changes seen in detached cells, EDTA treatment of cells could also influence the activity of several Mg2+-dependent metabolic enzymes. We therefore tested the effect of ouabain, an FDA-approved drug that has recently been shown to prevent clustering of cancer cells (Gkountela et al., 2019). Indeed, treating detached 293T cells with ouabain significantly reduced clustering (Figure S6E) and decreased M + 5 citrate and M + 3 malate (Figure S6F). These results mirrored those seen following EDTA treatment, further supporting the role of clustering in the metabolic adaptation. Utilizing a different approach, we allowed cells to form clusters following detachment before dissociating them into single cells by trypsin treatment. Strikingly, $^{13}C_5$-glutamine tracing revealed that reductive carboxylation into malate almost completely disappeared when cell clusters were dissociated into single cells (Figures 6E, 6F, and S6G), accompanied by a restoration of aspartate biosynthesis and FAO (Figure 6G), suggesting oxidative mitochondrial respiration. However, consistent with a previous report (Jiang et al., 2016), reductive carboxylation of glutamine into citrate was sustained in dissociated detached cells compared to attached cells—albeit to a lower level than seen in clustered detached cells (Figures 6E and S6G). The reactivation of mitochondrial oxidation seen in dissociated detached cells, together with our finding that detached cells have higher mitochondrial ROS production, suggested that these cells may accumulate increased mitochondrial ROS levels. Indeed, cells in which clustering was disrupted showed a higher production of mitochondrial ROS than cells allowed to form clusters (Figures 6H and S6H). Interestingly, detached cells that were allowed to form clusters showed a time-dependent decrease in mitochondrial ROS production (Figures 6H and S6H), likely reflecting the increase in cluster size and hypoxia over time. These data support a role for cell clustering in driving the metabolic switch that results in decreased mitochondrial metabolism, inability to carry out FAO, and decreased mitochondrial ROS production, and they show that this metabolic response can be reversed by disrupting cell clustering.

Preventing the Metabolic Switch during Detachment Increases Mitochondrial ROS and Limits Metastatic Capacity

To assess the importance of the metabolic switch toward glycolysis in detached cells, we replaced glucose with galactose in the culture media. Galactose is metabolized more slowly than glucose, and pyruvate production from galactose yields no net ATP production, compared to the 2 net ATP molecules yielded by glucose oxidation. Consequently, cells grown in galactose rely on OXPHOS for ATP production. Detached cells grown in galactose formed much smaller, looser clusters than cells grown in glucose (Figures 7A and S7A). Interestingly, clusters formed in galactose were less hypoxic than similar-sized clusters formed in glucose (Figures 7B and S7B) and had reduced expression of BNIP3 and NIX (Figure 7C). Consistently, less mitophagy was detected in detached cells grown in galactose compared to detached cells in grown in glucose (Figures 7D and S7C). In line with this, after 5 days in detached conditions, $^{13}C_5$-glutamine labeling revealed that detached cells grown in galactose did not make the switch to reductive glutamine metabolism and retained oxidative glutamine metabolism (Figures 7E and S7D). Also, aspartate levels were maintained at similar levels to those seen in attached cells, and there was no 2HG accumulation (Figures 7F and S7E). The maintenance of oxidative phosphorylation in detached cells grown in galactose corresponded to a significant increase in ROS production within 4 h after loss of attachment, increasing further over the subsequent 24 and 48 h (Figures 7G and S7F). These levels of ROS were much higher than those measured in detached cells grown in glucose, where mitochondrial oxidation was limited (Figure S7G). The increased oxidative stress in the detached cells grown in galactose was also demonstrated by a substantial reduction in the GSH/GSSG ratio

(F) Western blot analysis of detached 293T, HeLa, and A549 cells showing expression of HIF1α, NIX, BNIP3 and β-actin (NIX was detected on a duplicate blot) in the absence or presence of siHIF1. Equal protein amounts were loaded per condition and verified by ponceau staining. β-Actin was used to verify the integrity of the samples.

(G) HeLa cells were transfected with mitochondrial-targeted mKeima and cultured in attached or detached conditions for 3 days before flow cytometry analysis. Mitochondria in cytosol (pH 7) are represented by mKeima with excitation of 405 nm, while mitochondria in lysosomes (pH 4) are represented by mKeima with excitation of 561 nm. Mitophagy is reflected by the percentage of cells in the upper panel represented by the graph showing mean ± SD of triplicate cultures in a representative experiment.

(H) Mitochondrial mass of 293T and HeLa attached and detached cells were measured using Mito Tracker green fluorescent dye and analyzed by flow cytometry.

(I) Western blot analysis of MiaPACA, A549, SW480, MDA-MB-468, HeLa and 293T attached and detached cells showing expression of Tom20 and β-actin. Actin was detected on a duplicate blot. Equal protein amounts were loaded per condition and verified by ponceau staining. β-Actin was used to verify the integrity of the samples.

(J) Mitochondrial-targeted mkeima-expressing HeLa cells were cultured in detached conditions in the presence of non-targeting, Hif1α or BNIP3 + NIX siRNA and analyzed by flow cytometry for mitophagy as in (F).

(K) Mitochondrial mass of HeLa cells cultured in detached conditions in the presence of non-targeting, Hif1α or BNIP3 + NIX siRNA. Mitochondrial mass was measured using NAO staining and flow cytometry analysis.

(L) Mitochondrial ROS of HeLa cells cultured in detached conditions in the presence of non-targeting, Hif1α or BNIP3 + NIX siRNA measured by MitoSOX Red staining and flow cytometry analysis.

(G, H, J, K, and L) Data are presented as mean ± SD of triplicate cultures of representative experiments. $^{*}p \leq 0.05$, $^{**}p \leq 0.01$, $^{****}p \leq 0.0001$, unpaired t test.

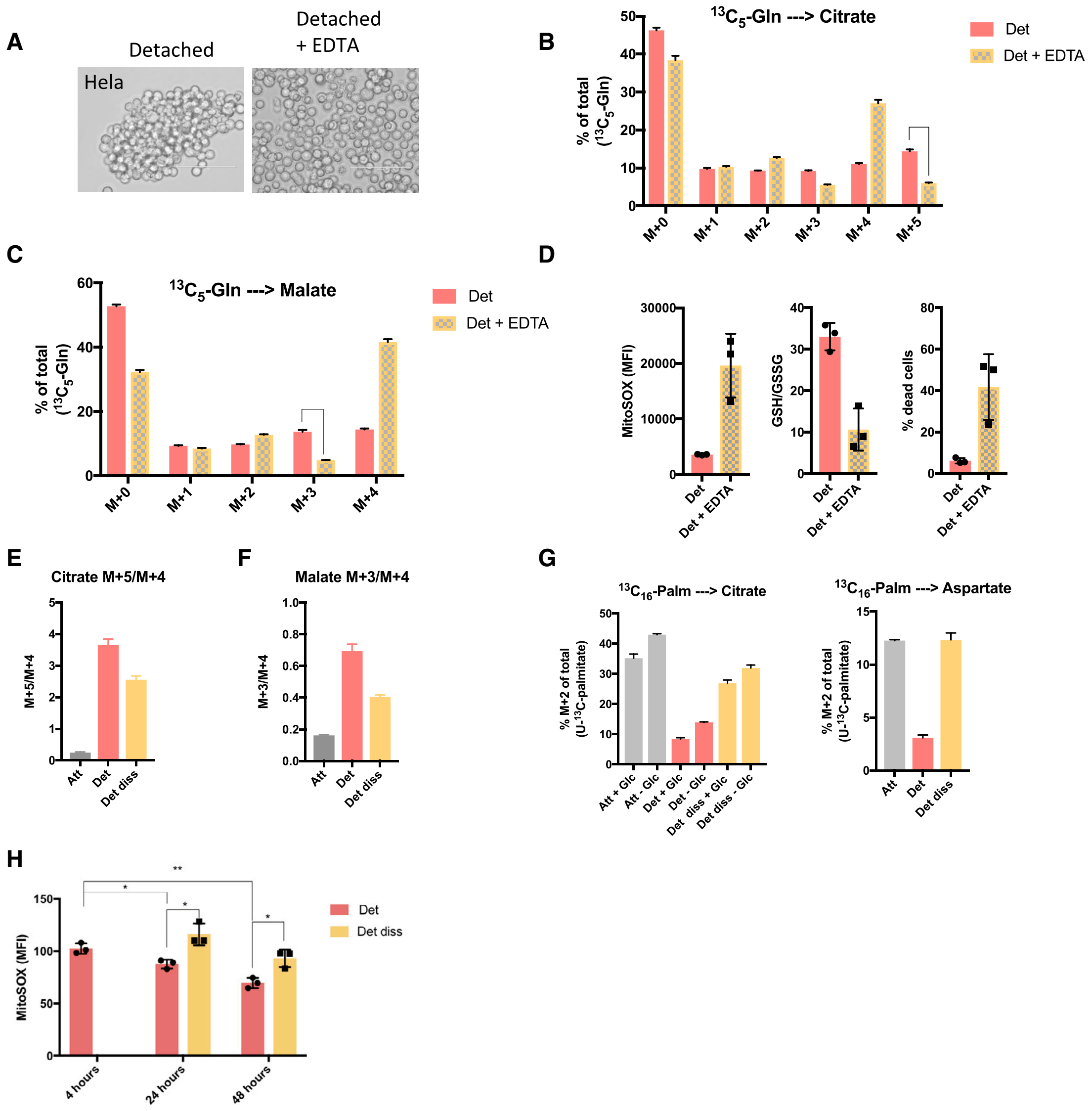

Figure 6. Cell Clustering Supports the Metabolic Switch and Reduces ROS in Detached Cells

(A) Representative images of HeLa cells treated with 2 mM EDTA to prevent clustering after matrix detachment. Cells were cultured for 48 h in detached conditions in the presence of EDTA. Scale bars represent 100 μm.

(B and C) Isotopomer distribution of citrate (B) and malate (C) in HeLa cells grown in detached conditions with or without EDTA.

(D) Preventing cell clustering after matrix detachment induce an increase in mitochondrial ROS production, oxidative stress, and cell death. HeLa cells were cultured in detached conditions with or without EDTA for 3 days before mitochondrial ROS, GSH/GSSG, and cell viability analysis.

(E and F) M + 5/M + 4 of citrate (E) and M + 3/M + 4 of malate (F) originating from $^{13}C_5$-glutamine in HeLa cells cultured in attached conditions, detached conditions and detached conditions before dissociation into single cells (Det diss).

(G) Fraction of M + 2 citrate (left panel) and aspartate (right panel) originating from $^{13}C_{16}$-palmitate in HeLa cells grown in attached conditions, detached conditions, and detached conditions before dissociation into single cells with or without glucose for 4 h.

(H) Levels of mitochondrial ROS production in HeLa cells cultured in detached conditions and detached conditions before dissociation into single cells for indicated times. Mitochondrial ROS was measured by staining cells with MitoSOX Red before analysis by flow cytometry.

(B–H) Data are represented as mean ± SD of triplicate cultures of representative experiments.

*p ≤ 0.05, **p ≤ 0.01. Unpaired t test.

(Figure 7H). There was no difference in cell viability in attached cells grown in either glucose or galactose, whereas detached cells in galactose showed an increase in cell death compared to cells grown in glucose (Figures 7I and S7H). Taken together, these results show that while detached cells normally switch to reductive metabolism and glycolysis, they retain the capacity for oxidative phosphorylation. However, when forced to use this pathway for ATP production, detached cells accumulate increased mitochondrial ROS and oxidative stress.

To determine whether this response to detachment contributes to the growth or dissemination of cancer cells *in vivo*, we examined tumor growth and metastatic capacity of cancer

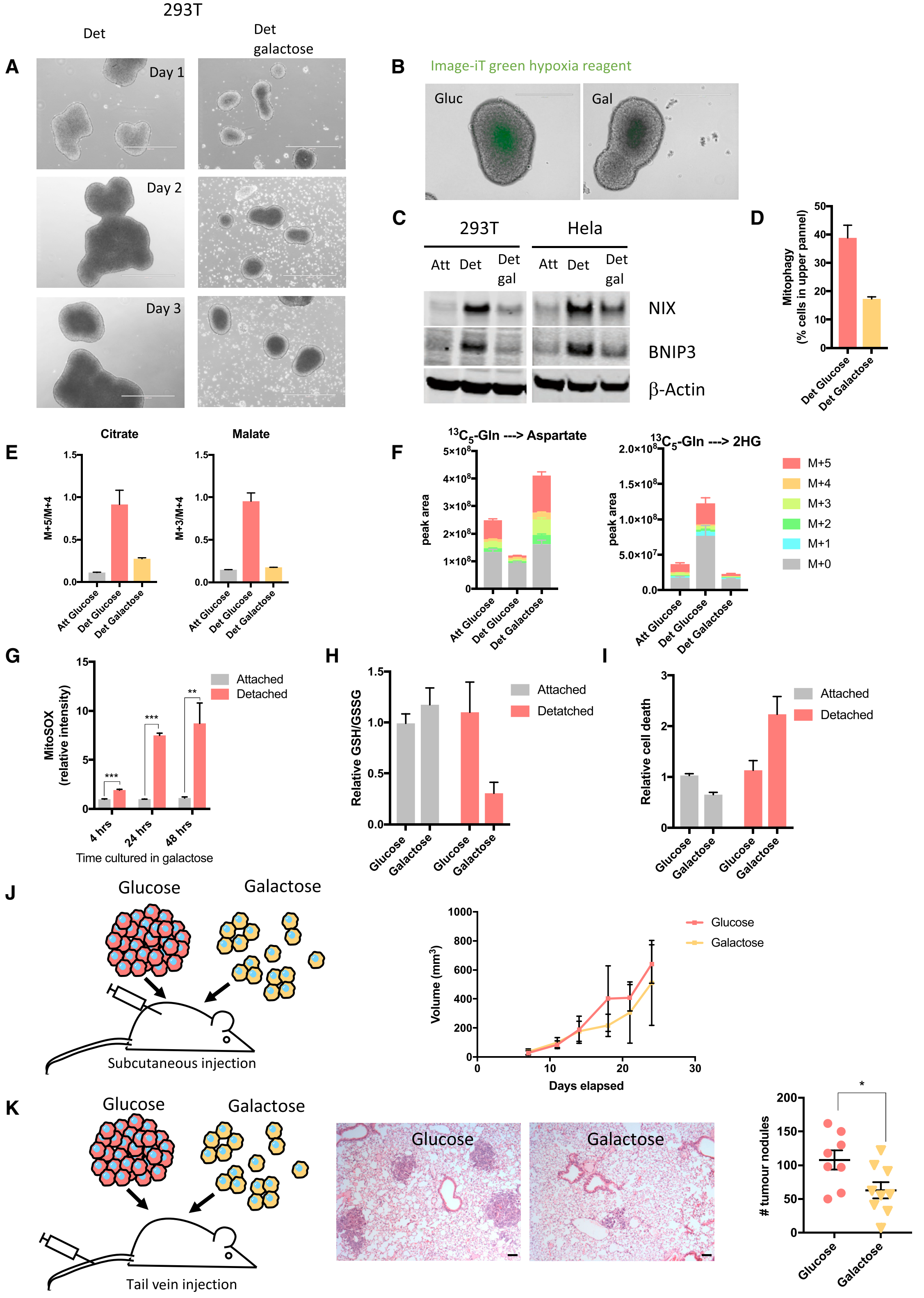

Figure 7. Metabolic Adaptation during Detachment Supports Metastatic Colonization

(A) Representative images of detached 293T cells grown in glucose (left panel) and galactose (right panel) for 3 days. Scale bars represent 1,000 μm.

(B) Image-iT green hypoxia reagent staining of detached 293T cells grown in glucose (left panel) or galactose (right panel) for 3 days before live cell staining was performed. Scale bars represent 400 μm.

(legend continued on next page)

cells that were grown in suspension in the presence of glucose (to allow clustering and metabolic adaptation) or galactose (to limit clustering and prevent metabolic adaptation). In order to assess these parameters of tumor behavior *in vivo* in the context of a functional immune system, we turned to cells derived from mouse pancreatic ductal adenocarcinoma driven by KRas and p53 mutations (Hingorani et al., 2005). As seen using the human cancer cells, detachment of these mouse tumor cells resulted in clustering and a switch to reductive metabolism that was prevented by growth in galactose (Figures S7I and S7J). The ability of these cells to form primary tumors was monitored after subcutaneous injection of detached cells, while metastatic colonization capacity was assessed by measuring tumor nodule formation in the lungs of mice following tail vain injection (Figures 7J and 7K). No significant difference in subcutaneous tumor growth was seen following injection of detached cells grown in glucose or galactose (Figure 7J), indicating that the formation of a tumor mass provided sufficient support to allow cell survival and proliferation. By contrast, cells grown in galactose showed a significantly reduced ability to form lung colonies following tail vein injection (Figure 7K), supporting the proposal that metabolic adaptation induced by cell clustering promotes the ability of cancer cells to survive in the circulation and disseminate to distant sites.

DISCUSSION

Several previous studies have shown that both ROS regulation (Jiang et al., 2016; Schafer et al., 2009) and cell clustering (Aceto et al., 2014; Gkountela et al., 2019; Liu et al., 2019) can promote metastasis, and in this study, we show that these responses are linked through a metabolic adaptation that promotes cell survival. We show that loss of attachment has a profound impact on mitochondrial morphology and function, leading to an increase in mitochondrial ROS production. Cell survival depends on the ability to form clusters following detachment, driving the induction of Hif1α-mediated mitophagy to remove ROS-generating, damaged mitochondria. A previous report showed that Hif1α can also mediate anoikis resistance through suppression of alpha5 integrin (Rohwer et al., 2008), further supporting the advantage of clustering-induced hypoxia during matrix detachment.

Our data show that cell clustering induces a hypoxic environment, consistent with previous reports showing that tumor spheroids have hypoxic centers (Riffle and Hegde, 2017). This hypoxic environment leads to stabilization of Hif1α, which drives mitophagy via BNIP3 and NIX. Mitophagy is associated with clearance of damaged mitochondria and consequently helps to limit mitochondrial ROS. Indeed, we show that disruption of the Hif1α-BNIP3/NIX pathway leads to a decrease in mitophagy and accumulation of mitochondrial ROS in detached cells. Interestingly, a recent report showed that loss of attachment induces RIPK1-mediated mitophagy in MCF10A cells that lead to decreased cell viability by reducing mitochondrial IDH2-mediated NADPH production. Many cancer cell lines, however, express RIPK1 in attached and detached conditions and possibly develop a capacity to restrict RIPK1-mediated cell death (Hawk et al., 2018).

While the hypoxia-mediated elimination of damaged mitochondria protects cells from ROS-induced death, a consequence of this adaptation is a switch from mitochondrial metabolism to glycolysis. As a result, detached cells become dependent on glycolysis for ATP production and survival and are unable to use alternative nutrient sources that require OXPHOS for ATP production. These observations suggest a possible vulnerability of detached, metastasizing cells that could be targeted for therapy.

Our data show that cell clustering-mediated hypoxia is in part responsible for the metabolic switch we detect in detached cells. Hypoxia induces reductive carboxylation and dependence on glucose for ATP production (Eales et al., 2016), consistent with our observations in detached cells. Dissociation of cell clusters into single cells largely reverse the metabolic switch to oxidative mitochondrial metabolism, resulting in more mitochondrial ROS production. Importantly, although dissociation into single cells almost completely eliminated reductive carboxylation of glutamine into malate, it reduced but did not eliminate reductive carboxylation into citrate. This observation is consistent with previous work showing that this citrate can be used to produce mitochondrial NADPH to limit ROS (Jiang et al., 2016). Taken together, the induction of

(C) Western blot analysis of attached and detached 293T and HeLa cells grown in glucose or galactose showing expression of NIX, BNIP3, and β-actin (NIX was detected on a duplicate blot). Equal protein amounts were loaded per condition and verified by ponceau staining. β-actin was used to verify the integrity of the samples.

(D) Mitochondrial-targeted mkeima-expressing HeLa cells were cultured in detached conditions in glucose or galactose and analyzed by flow cytometry for mitophagy.

(E) $^{13}C_5$-labeled glutamine-derived M + 5/M + 4 citrate and M + 3/M + 4 malate of 293T cells grown in attached and detached conditions with media containing either glucose or galactose.

(F) Levels and isotopomer distribution of intracellular aspartate and 2-HG of 293T cells cultured in the same conditions as (E). Peak area levels are normalized to cell number.

(G) Mitochondrial ROS analyzed using MitoSOX Red dye in attached and detached 293T cells grown in galactose. Cells were cultured in attached or detached conditions with galactose for indicated times before MitoSOX Red staining and analysis by flow cytometry.

(H) GSH/GSSG in 293T attached and detached cells grown in either glucose or galactose for 5 days.

(I) Fraction of dead 293T cells grown in attached or detached conditions with either glucose or galactose.

(J) Schematic illustration of experimental setup and tumor volume following subcutaneous injection of detached cells grown in glucose or galactose. Error bars represent the SD.

(K) Schematic illustration of experimental setup, representative images of lung H&E staining, and quantification of lung tumor nodules following tail vein injection of detached cells grown in glucose or galactose. Scale bars represent 10 μM. Error bars are SEM. * $p \leq 0.05$, unpaired t test.

(D–I) Data are presented as mean ± SD of triplicate cultures of representative experiments.

** $p \leq 0.01$, *** $p \leq 0.001$, **** $p \leq 0.0001$ (G), multiple t test.

reductive glutamine metabolism in response to detachment appears to contribute to the support of two distinct responses that limits mitochondrial ROS production. We also note that in some cells, induction of membrane ROS through NADPH oxidase 4 can induce EGFR and thus protect from anoikis (Du et al., 2018). Previous reports have shown that inhibition of cluster formation by depletion of adhesion proteins leads to a diminished metastatic capacity (Aceto et al., 2014; Cheung et al., 2016b; Liu et al., 2019). We also show that detached cells upregulate cadherin expression, which is likely to contribute to the ability to cluster. Interestingly, however, cells that are grown in galactose and are unable to make the metabolic switch to glycolysis are defective in cluster formation. Whether the metabolic switch promotes the expression of adhesion proteins or whether growth in galactose selects for cells with a low adhesive capacity remains to be determined. Interestingly, we show that the properties of cells grown in galactose *in vitro* are maintained to the extent that metastatic capacity is reduced when placed *in vivo*. We speculate that this "memory" may reflect changes in DNA or chromatin modification resulting from the increased L-2HG accumulation in these cells. Our data show that cell survival following detachment depends on cell clustering and hypoxia-induced mitophagy, which limits high mitochondrial ROS by removing the damaged mitochondria that are found in detached cells. By contrast, attached cells do not accumulate damaged mitochondria and so do not depend on this metabolic remodeling for survival. While care must be taken in extrapolating our results, which were generated with cell lines in different culture models, they highlight a potential difference between cells in normal tissue, where interactions with ECM prevent the acquisition of mitochondrial damage and increased ROS, and the cell-cell adhesion that promotes cell clustering and a metabolic switch to circumvent the consequences of detachment and may be experienced once cancer cells have left the primary tumor site and disseminate through the bloodstream. While the ability of cells to invade out of the primary tumor can be supported by interaction with a stiffer ECM (He et al., 2016), our work suggests that targeting the metabolic changes that promote clustering-induced survival will retard some of the subsequent stages of metastatic spread.

Limitations of Study

Our work focuses on tumor cell lines, mostly studied in tissue culture. *In vivo* models included subcutaneous and tail vein injections into nude mice. The progression of spontaneous tumors in an immunocompetent host is likely to be more complex than our model systems.

STAR★METHODS

Detailed methods are provided in the online version of this paper and include the following:

- KEY RESOURCES TABLE
- LEAD CONTACT AND MATERIALS AVAILABILITY
- EXPERIMENTAL MODEL AND SUBJECT DETAILS
    - Cell Culture
    - Mice
- METHOD DETAILS
    - Stable Isotope Tracing and Liquid Chromatography-Mass Spectrometry
    - Chiral Separation of L- and D-2-Hydroxyglutarate by GC-MS
    - Western Blotting
    - siRNA Transfection
    - Oxygen Consumption Rates
    - Flow Cytometry
    - Imaging
    - *In Vivo* Experiments
    - Immunohistochemistry
- QUANTIFICATION AND STATISTICAL ANALYSIS
- DATA AND CODE AVAILABILITY

SUPPLEMENTAL INFORMATION

Supplemental Information can be found online at https://doi.org/10.1016/j.cmet.2019.07.014.

ACKNOWLEDGMENTS

We would like to thank the Biological Research Facility, experimental histopathology and metabolomics, at the Crick for assistance with the work. This work was funded by Cancer Research UK grants C596/A10419 and C596/A26855 and supported by the Francis Crick Institute, which receives its core funding from Cancer Research UK (FC0010557), the UK Medical Research Council (FC0010557), and the Wellcome Trust (FC0010557).

AUTHOR CONTRIBUTIONS

The study was conceived and designed by C.F.L. and K.H.V. Experiments were conducted by C.F.L., E.C.C., and J.B. SEM was carried out in collaboration with M.-C.D. All authors contributed to writing and editing the manuscript.

DECLARATION OF INTERESTS

K.H.V. is on the Board of Directors and is a shareholder of Bristol Myers Squibb, is a shareholder of GRAIL Inc., and is on the Science Advisory Board of PMV Pharma, RAZE Therapeutics, and Ludwig Cancer. She is a founder of and consultant for Faeth Therapeutics. She has been in receipt of research funding from Astex Pharmaceuticals and is in receipt of research funding from AstraZeneca. She contributed to CRUK Cancer Research Technology filing of Patent Application WO/2017/144877.

Received: March 26, 2019
Revised: April 30, 2019
Accepted: July 29, 2019
Published: August 22, 2019

REFERENCES

Aceto, N., Bardia, A., Miyamoto, D.T., Donaldson, M.C., Wittner, B.S., Spencer, J.A., Yu, M., Pely, A., Engstrom, A., Zhu, H., et al. (2014). Circulating tumor cell clusters are oligoclonal precursors of breast cancer metastasis. Cell *158*, 1110–1122.

Birsoy, K., Wang, T., Chen, W.W., Freinkman, E., Abu-Remaileh, M., and Sabatini, D.M. (2015). An essential role of the mitochondrial electron transport chain in cell proliferation is to enable aspartate synthesis. Cell *162*, 540–551.

Brown, C.W., Amante, J.J., Goel, H.L., and Mercurio, A.M. (2017). The α6β4 integrin promotes resistance to ferroptosis. J. Cell Biol. *216*, 4287–4297.

Brown, C.W., Amante, J.J., and Mercurio, A.M. (2018). Cell clustering mediated by the adhesion protein PVRL4 is necessary for α6β4 integrin-promoted ferroptosis resistance in matrix-detached cells. J. Biol. Chem. *293*, 12741–12748.

Buchheit, C.L., Weigel, K.J., and Schafer, Z.T. (2014). Cancer cell survival during detachment from the ECM: multiple barriers to tumour progression. Nat. Rev. Cancer *14*, 632–641.

Cheung, E.C., Lee, P., Ceteci, F., Nixon, C., Blyth, K., Sansom, O.J., and Vousden, K.H. (2016a). Opposing effects of TIGAR- and RAC1-derived ROS on Wnt-driven proliferation in the mouse intestine. Genes Dev. *30*, 52–63.

Cheung, K.J., Padmanaban, V., Silvestri, V., Schipper, K., Cohen, J.D., Fairchild, A.N., Gorin, M.A., Verdone, J.E., Pienta, K.J., Bader, J.S., et al. (2016b). Polyclonal breast cancer metastases arise from collective dissemination of keratin 14-expressing tumor cell clusters. Proc. Natl. Acad. Sci. USA *113*, E854–E863.

Chourasia, A.H., Tracy, K., Frankenberger, C., Boland, M.L., Sharifi, M.N., Drake, L.E., Sachleben, J.R., Asara, J.M., Locasale, J.W., Karczmar, G.S., et al. (2015). Mitophagy defects arising from BNip3 loss promote mammary tumor progression to metastasis. EMBO Rep. *16*, 1145–1163.

Deerinck, T.J., Bushong, E., Thor, A., and Ellisman, M.H. (2010). NCMIR methods for 3D EM: a new protocol for preparation of biological specimens for serial block-face SEM. Microscopy *6–8*.

Du, S., Miao, J., Zhu, Z., Xu, E., Shi, L., Ai, S., Wang, F., Kang, X., Chen, H., Lu, X., et al. (2018). NADPH oxidase 4 regulates anoikis resistance of gastric cancer cells through the generation of reactive oxygen species and the induction of EGFR. Cell Death Dis. *9*, 948.

Eales, K.L., Hollinshead, K.E.R., and Tennant, D.A. (2016). Hypoxia and metabolic adaptation of cancer cells. Oncogenesis *5*, e190.

Eccles, S.A., and Welch, D.R. (2007). Metastasis: recent discoveries and novel treatment strategies. Lancet *369*, 1742–1757.

Fidler, I.J. (2003). The pathogenesis of cancer metastasis: the "seed and soil" hypothesis revisited. Nat. Rev. Cancer *3*, 453–458.

Gaude, E., Schmidt, C., Gammage, P.A., Dugourd, A., Blacker, T., Chew, S.P., Saez-Rodriguez, J., O'Neill, J.S., Szabadkai, G., Minczuk, M., et al. (2018). NADH shuttling couples cytosolic reductive carboxylation of glutamine with glycolysis in cells with mitochondrial dysfunction. Mol. Cell *69*, 581–593.e7.

Gkountela, S., Castro-Giner, F., Szczerba, B.M., Vetter, M., Landin, J., Scherrer, R., Krol, I., Scheidmann, M.C., Beisel, C., Stirnimann, C.U., et al. (2019). Circulating tumor cell clustering shapes DNA methylation to enable metastasis seeding. Cell *176*, 98–112.e14.

Goh, J., Enns, L., Fatemie, S., Hopkins, H., Morton, J., Pettan-Brewer, C., and Ladiges, W. (2011). Mitochondrial targeted catalase suppresses invasive breast cancer in mice. BMC Cancer *11*, 191.

Hanse, E.A., Ruan, C., Kachman, M., Wang, D., Lowman, X.H., and Kelekar, A. (2017). Cytosolic malate dehydrogenase activity helps support glycolysis in actively proliferating cells and cancer. Oncogene *36*, 3915–3924.

Hawk, M.A., Gorsuch, C.L., Fagan, P., Lee, C., Kim, S.E., Hamann, J.C., Mason, J.A., Weigel, K.J., Tsegaye, M.A., Shen, L., et al. (2018). RIPK1-mediated induction of mitophagy compromises the viability of extracellular-matrix-detached cells. Nat. Cell Biol. *20*, 272–284.

Hawk, M.A., and Schafer, Z.T. (2018). Mechanisms of redox metabolism and cancer cell survival during extracellular matrix detachment. J. Biol. Chem. *293*, 7531–7537.

He, X., Lee, B., and Jiang, Y. (2016). Cell-ECM interactions in tumor invasion. Adv. Exp. Med. Biol. *936*, 73–91.

Hingorani, S.R., Wang, L., Multani, A.S., Combs, C., Deramaudt, T.B., Hruban, R.H., Rustgi, A.K., Chang, S., and Tuveson, D.A. (2005). Trp53R172H and KrasG12D cooperate to promote chromosomal instability and widely metastatic pancreatic ductal adenocarcinoma in mice. Cancer Cell *7*, 469–483.

Huang, D., Li, T., Li, X., Zhang, L., Sun, L., He, X., Zhong, X., Jia, D., Song, L., Semenza, G.L., et al. (2014). HIF-1-mediated suppression of acyl-CoA dehydrogenases and fatty acid oxidation is critical for cancer progression. Cell Rep. *8*, 1930–1942.

Humpton, T.J., Alagesan, B., DeNicola, G.M., Lu, D., Yordanov, G.N., Leonhardt, C.S., Yao, M.A., Alagesan, P., Zaatari, M.N., Park, Y., et al. (2019). Oncogenic Kras induces Nix-mediated mitophagy to promote pancreatic cancer. Cancer Discov.

Hunter, K.W., Crawford, N.P.S., and Alsarraj, J. (2008). Mechanisms of metastasis. Breast Cancer Res. *10*, S2.

Intlekofer, A.M., Dematteo, R.G., Venneti, S., Finley, L.W.S., Lu, C., Judkins, A.R., Rustenburg, A.S., Grinaway, P.B., Chodera, J.D., Cross, J.R., et al. (2015). Hypoxia induces production of L-2-hydroxyglutarate. Cell Metab. *22*, 304–311.

Intlekofer, A.M., Wang, B., Liu, H., Shah, H., Carmona-Fontaine, C., Rustenburg, A.S., Salah, S., Gunner, M.R., Chodera, J.D., Cross, J.R., et al. (2017). L-2-Hydroxyglutarate production arises from noncanonical enzyme function at acidic pH. Nat. Chem. Biol. *13*, 494–500.

Jiang, L., Shestov, A.A., Swain, P., Yang, C., Parker, S.J., Wang, Q.A., Terada, L.S., Adams, N.D., McCabe, M.T., Pietrak, B., et al. (2016). Reductive carboxylation supports redox homeostasis during anchorage-independent growth. Nature *532*, 255–258.

Kubli, D.A., and Gustafsson, Å.B. (2012). Mitochondria and mitophagy: the Yin and Yang of cell death control. Circ. Res. *111*, 1208–1221.

Labuschagne, C.F., van den Broek, N.F., Mackay, G.M., Vousden, K.H., and Maddocks, O.K. (2014). Serine, but not glycine, supports one-carbon metabolism and proliferation of cancer cells. Cell Rep. *7*, 1248–1258.

Lazarou, M., Sliter, D.A., Kane, L.A., Sarraf, S.A., Wang, C., Burman, J.L., Sideris, D.P., Fogel, A.I., and Youle, R.J. (2015). The ubiquitin kinase PINK1 recruits autophagy receptors to induce mitophagy. Nature *524*, 309–314.

Le Gal, K., Ibrahim, M.X., Wiel, C., Sayin, V.I., Akula, M.K., Karlsson, C., Dalin, M.G., Akyürek, L.M., Lindahl, P., Nilsson, J., and Bergo, M.O. (2015). Antioxidants can increase melanoma metastasis in mice. Science Translational Medicine *7*, 308re8.

Lewis, C.A., Parker, S.J., Fiske, B.P., McCloskey, D., Gui, D.Y., Green, C.R., Vokes, N.I., Feist, A.M., Vander Heiden, M.G., and Metallo, C.M. (2014). Tracing compartmentalized NADPH Metabolismin the cytosol and mitochondria of mammalian cells. Mol. Cell *55*, 253–263.

Li, T., Fan, J., Wang, B., Traugh, N., Chen, Q., Liu, J.S., Li, B., and Liu, X.S. (2017). TIMER: A web server for comprehensive analysis of tumor-infiltrating immune cells. Cancer Res. *77*, e108–e110.

Liu, X., Taftaf, R., Kawaguchi, M., Chang, Y.F., Chen, W., Entenberg, D., Zhang, Y., Gerratana, L., Huang, S., Patel, D.B., et al. (2019). Homophilic CD44 interactions mediate tumor cell aggregation and polyclonal metastasis in patient-derived breast cancer models. Cancer Discov. *9*, 96–113.

Maître, J.L., and Heisenberg, C.P. (2013). Three functions of cadherins in cell adhesion. Curr. Biol. *23*, R626–R633.

Mullen, A.R., Wheaton, W.W., Jin, E.S., Chen, P.H., Sullivan, L.B., Cheng, T., Yang, Y., Linehan, W.M., Chandel, N.S., and DeBerardinis, R.J. (2011). Reductive carboxylation supports growth in tumour cells with defective mitochondria. Nature *481*, 385–388.

Piskounova, E., Agathocleous, M., Murphy, M.M., Hu, Z., Huddlestun, S.E., Zhao, Z., Leitch, A.M., Johnson, T.M., DeBerardinis, R.J., and Morrison, S.J. (2015). Oxidative stress inhibits distant metastasis by human melanoma cells. Nature *527*, 186–191.

Place, T.L., Domann, F.E., and Case, A.J. (2017). Limitations of oxygen delivery to cells in culture: an underappreciated problem in basic and translational research. Free Radic. Biol. Med. *113*, 311–322.

Porporato, P.E., Payen, V.L., Pérez-Escuredo, J., De Saedeleer, C.J., Danhier, P., Copetti, T., Dhup, S., Tardy, M., Vazeille, T., Bouzin, C., et al. (2014). A mitochondrial switch promotes tumor metastasis. Cell Rep. *8*, 754–766.

Riffle, S., and Hegde, R.S. (2017). Modeling tumor cell adaptations to hypoxia in multicellular tumor spheroids. J. Exp. Clin. Cancer Res. *36*, 102.

Rohwer, N., Welzel, M., Daskalow, K., Pfander, D., Wiedenmann, B., Detjen, K., and Cramer, T. (2008). Hypoxia-inducible factor 1alpha mediates anoikis resistance via suppression of alpha5 integrin. Cancer Res. *68*, 10113–10120.

Sauvat, A., Wang, Y., Segura, F., Spaggiari, S., Müller, K., Zhou, H., Galluzzi, L., Kepp, O., and Kroemer, G. (2015). Quantification of cellular viability by automated microscopy and flow cytometry. Oncotarget *6*, 9467–9475.

Schafer, Z.T., Grassian, A.R., Song, L., Jiang, Z., Gerhart-Hines, Z., Irie, H.Y., Gao, S., Puigserver, P., and Brugge, J.S. (2009). Antioxidant and oncogene rescue of metabolic defects caused by loss of matrix attachment. Nature *461*, 109–113.

Simpson, C.D., Anyiwe, K., and Schimmer, A.D. (2008). Anoikis resistance and tumor metastasis. Cancer Lett. *272*, 177–185.

Sood, A., Jeyaraju, D.V., Prudent, J., Caron, A., Lemieux, P., McBride, H.M., Laplante, M., Tóth, K., and Pellegrini, L. (2014). A mitofusin-2-dependent inactivating cleavage of Opa1 links changes in mitochondria cristae and ER contacts in the postprandial liver. Proc. Natl. Acad. Sci. USA *111*, 16017–16022.

Sowter, H.M., Ratcliffe, P.J., Watson, P., Greenberg, A.H., and Harris, A.L. (2001). HIF-1-dependent regulation of hypoxic induction of the cell death factors BNIP3 and NIX in human tumors. Cancer Res. *61*, 6669–6673.

Sullivan, L.B., Gui, D.Y., Hosios, A.M., Bush, L.N., Freinkman, E., and Vander Heiden, M.G. (2015). Supporting aspartate biosynthesis is an essential function of respiration in proliferating cells. Cell *162*, 552–563.

Zhang, H., Bosch-Marce, M., Shimoda, L.A., Tan, Y.S., Baek, J.H., Wesley, J.B., Gonzalez, F.J., and Semenza, G.L. (2008). Mitochondrial autophagy is an HIF-1-dependent adaptive metabolic response to hypoxia. J. Biol. Chem. *283*, 10892–10903.

STAR★METHODS

KEY RESOURCES TABLE

REAGENT or RESOURCE	SOURCE	IDENTIFIER
Antibodies		
Rabbit polyclonal anti-Pyruvate Dehydrogensae E1-alpha subunit (phospho S293)	Abcam	Cat#ab92696
Mouse monoclonal anti-Pyruvate Dehydrogenase E1-alpha subunit	Abcam	Cat#ab110334
Mouse monoclonal anti-beta Actin	Abcam	Cat#ab8226
Rabbit monoclonal antiMDH1	Abcam	Cat#ab180152
Mouse polyclonal anti-LDHA	Abcam	Cat#ab92903
Rabbit polyclonal anti-Pan-Cadherin	Cell Signaling Technology	Cat#4068T
Monoclonal Rabbit anti-Hif1α (D2U3T)	Cell Signaling Technology	Cat#14179S
Monoclonal Rabbit anti-BNIP3L/Nix (D4R4B)	Cell Signaling Technology	Cat#12396S
Monoclonal Rabbit anti-BNIP3 (D7U1T)		N/A
Monoclonal Mouse anti-Tom20	Santa Cruz Biotechnology	Cat#sc-136211
Chemicals, Peptides, and Recombinant Proteins		
D-Glucose (U-13C6, 99%)	Cambridge Isotope Laboratories	Cat#CLM-1396; CAS: 110187-42-3
D-Glucose (4-D, 98%)	Cambridge Isotope Laboratories	Cat#DLM-9294-PK; CAS: 56570-89-9
Palmitic acid (U-13C16, 98%)	Cambridge Isotope Laboratories	Cat#CLM-409-0.5;
L-Glutamine (13C5, 98 atom % 13C, 95% (CP))	Sigma-Aldrich	Cat#605166; CAS:184161-19-1
Corning™ Cell-Tak Cell and Tissue Adhesive	Fisher Scientific	Cat#10317081
MitoSOX™ Red	Thermo Fisher Scientific	Cat#M36008
DAPI	Thermo Fisher Scientific	Cat#D1306
MitoTreacker™ Green FM	Thermo Fisher Scientific	Cat#M7514
Nonyl Acridine Orange (NAO)	Thermo Fisher Scientific	Cat#A1372
eBioscience™ Fixable Viability Dye eFluor™780	Thermo Fisher Scientific	Cat#65-0865-14
Image-iT™ Green Hypoxia Reagent	Thermo Fisher Scientific	Cat#I14834
BODIPY™558/568 C12	Thermo Fisher Scientific	Cat#D3835
EDTA	Sigma-Aldrich	Cat#E5134
Ouabain	Sigma-Aldrich	Cat#PHR1945
Critical Commercial Assays		
Seahorse XF Cell Mito Stress Test Kit	Agilent	Cat#103015-100
Experimental Models: Cell Lines		
Human: 293T	The Francis Crick – Cell services	N/A
Human: A549	ATCC	CCL-185
Human: HeLa	ATCC	CCL-2
Human: SW480	ATCC	CCL-228
Human: Mia-PaCa-2	ATCC	CRL-1420
Human: MDA-MB-468	The Francis Crick - Cell Services	N/A
Human: HeLa mito YFP	A gift from Dr. Stephan Tait, Cancer research UK Beatson Institute	N/A
Mouse: KPC FC1242 PDAC cells	The Tuveson lab	Hingorani et al., 2005
Experimental Models: Organisms/Strains		
Mouse: Athymic Nude, nu/nu	The Jackson Laboratory	002019
Mouse: C57BL16J	The Jackson Laboratory	N/A

(*Continued on next page*)

Continued

REAGENT or RESOURCE	SOURCE	IDENTIFIER
Oligonucleotides		
SMARTpool:siGENOME Human MDH1 siRNA	Dharmacon	M-009264-00-0005
SMARTpool:siGENOME Human MDH2 siRNA	Dharmacon	M-008439-00-0005
SMARTpool:siGENOME Human LDHA	Dharmacon	M-008201-01-0005
SMARTpool:ON-TARGET plus Human Hιφ1α	Dharmacon	L-004018-00-0005
SMARTpool:ON-TARGET plus Human BNIP3L	Dharmacon	L-011815-00-0005
SMARTpool:ON-TARGET plus Human BNIP3	Dharmacon	L-004636-00-0005
siGENOME Non-Targeting siRNA Pool#2	Dharmacon	D-001206-14-20
Recombinant DNA		
pCHAC-mt-mKeima	Lazarou et al., 2015	Addgene Plasmid #72342
Software and Algorithms		
GraphPad Prism 7	GraphPad software	N/A
FlowJo software v.10.3	FlowJo	N/A
TraceFinder Version 4.1	Thermo Fisher Scientific	OPTON-30626
ImageJ	https://doi.org/10.1038/nmeth.2089	https://imagej.nih.gov/ij/
ZEN blue	Zeiss	https://www.zeiss.com/microscopy/int/products/microscope-software/zen-lite.html

LEAD CONTACT AND MATERIALS AVAILABILITY

Further information and requests for resources and reagents should be directed to and will be fulfilled by the Lead Contact, Karen H. Vousden (karen.vousden@crick.ac.uk).

EXPERIMENTAL MODEL AND SUBJECT DETAILS

Cell Culture

HeLa cells expressing YFP targeted to the mitochondrial matrix were a gift from Dr. Stephan Tait. KPC FC1242 mouse pancreatic ductal adenocarcinoma (PDAC) cells were a gift from the Tuveson lab isolated from PDAC tumors of LSL-Kras^{G12D}; LSL-TYrp53^{R172H},Pdx1-Cre mice (Hingorani et al., 2005). Stock cells of 293T (fetus), A549 (male), HeLa (female), SW480 (male), MIA PaCa-2 (male) and MDA-MB-468 (female) were maintained in DMEM (Thermo Fisher Scientific, 41966) supplemented with 10% FBS. All cells were cultured in a humidified atmosphere at 37°C with 5% CO_2. Cells grown in attached conditions were cultured in 6 well plates while cells grown in detached conditions were cultured in ultra-low adherent 6 well plates for indicated times. Cells in attached and detached conditions were cultured in identical media which were refreshed every day. For detached conditions cells were seeded at 2 x 10^5 per well in ultra-low attachment 6 well plates and cultured for 5 days before experiments were performed unless stated otherwise. For attached condition 1 X 10^5 cells were seeded per well in a 6 well plate and cultured for 2 days before experiments were performed. For glucose starvation experiments cells were cultured in glucose free DMEM supplemented with 10% dialysed FBS. For experiments where cells were grown in galactose, glucose free DMEM were supplemented with 12.5 mM galactose and 10% FBS. To prevent cell clustering in detached cells, cells were treated with EDTA or Ouabain.

Mice

All animal experiments were performed under the Animals (Scientific Procedures) Act 1986 and the EU Directive 2010 and sanctioned by local ethical review process (Francis Crick Institute). For tumor growth experiments C57BL16J female mice (obtained from The Jackson Laboratory, 6 weeks old) were used. For the lung metastasis model athymic *nu/nu* female mice (obtained from The Jackson Laboratory, 6 weeks old) were used. 5 mice were housed per cage in a room with a constant temperature(19-23°C) and humidity (55% ± 10%)and a 12-hour light/dark cycle (lights on at 7:00 am). Mice were allowed access to food and water *ad libitum*. Mice were randomly assigned to experimental groups after they were allowed to acclimatize for one week prior to the experiment.

METHOD DETAILS

Stable Isotope Tracing and Liquid Chromatography-Mass Spectrometry

Glucose, glutamine and palmitate labelled with ^{13}C and 4-^{2}H-glucose were purchased from Cambridge Isotope Laboratories. For isotopomer distribution studies of metabolites stable isotope tracing of $^{13}C_6$-glucose, $^{13}C_5$-glutamine and $^{13}C_{16}$-palmitate were

performed. Cells were cultured in attached or detached conditions as described above before media was changed with DMEM containing the labelled nutrients. For glucose tracing, media were replaced with glucose free DMEM supplemented with 25 mM $^{13}C_6$-glucose and 10% dialysed FBS for 4 hours before cells were washed twice with PBS and metabolites were extracted. For hydrogen tracing DMEM were supplemented with 25 mM 4-2H-glucose and 10% FBS for 4 hours before extraction. For glutamine tracing media were replaced by DMEM containing 2 mM $^{13}C_5$-glutamine and 10% dialysed FBS for 4 hours before metabolite extraction. Palmitate labelling was performed by replacing media with glucose free or glucose containing DMEM containing 100 μM $^{13}C_{16}$-palmitate and 1% FBS. Cells were cultured in the presence of $^{13}C_{16}$-palmitate for 4 hours before cells were washed 3 times with PBS and metabolites were extracted. Parallel wells were prepared to determine cell number for each condition which were used for normalisation. Extracted metabolites were analysed by LC-MS as described before (Labuschagne et al., 2014). Briefly, after cells were washed with PBS metabolites were extracted using ice cold extraction buffer consisting of methanol, acetonitrile and H_2O (50:30:20). Cell number was used to determine the volume of extraction buffer for each condition. Samples were then centrifuged at 16,000 x g for 10 minutes at 4°C and the supernatant were collected for LC-MS analysis.

To extract extracellular metabolites 10 μl of cell culture media were added to 490 μl of ice-cold extraction buffer and vortexed before samples were centrifuged as mentioned above. Supernatant were used for LC-MS analysis.

LC-MS was performed on a Dionex Ultimate 3000 LC system coupled to a Q Exactive mass spectrometer (Thermo Scientific). A Sequant ZIC-pHILIC column (2.1 x 150 mm, 5 uM) (Merck) was used to separate metabolites. Mobile phases consisted of 20 mM $(NH_4)_2CO_3$, 0.1% NH_4OH in H_2O (Buffer A) and acetonitrile (Buffer B). The flow rate was 200 ul/min with a gradient starting at 80% (A) decreasing to 20% (A) over 17 minutes followed by washing and re-equilibration steps. Ionization was achieved in a HESI probe connected to the Q Exactive which scanned a mass range between 75 and 1000 m/z with polarity switching. The acquired spectra were analysed using Thermo TraceFinder software.

Chiral Separation of L- and D-2-Hydroxyglutarate by GC-MS

Metabolites were extracted with 300 μl ice cold extraction buffer as described above and dried under a stream of nitrogen. The dried extract was resuspended in 150 μl (R)-2-butanol with 30 μl 1M HCl and heated for 1 hour at 100°C followed by drying under a stream of nitrogen gas. The dried extract was resuspended in 1:1 pyridine/acetic anhydride and heated for 30 min at 100°C again followed by drying under nitrogen. The final product was resuspended in chloroform and analysed by an Agilent 7890B GC system coupled to a 7000 Triple Quadropole mass spectrometry system. The column used was a Phenomonex 2B-1701 column (30m x 0,25 mm x 0.25 μm). The oven temperature programme used was 100°C for 3 minutes, 4°C per minute from 180°C to 230°C, 15°C per minute from 230°C-300°C and 300°C for 5 minutes.

Western Blotting

Whole cell protein lysates were prepared in RIPA buffer (Merck Millipore) containing 0.1% SDS, Pierce™ protease inhibitors (Thermo Scientific) and Halt™ phosphatase inhibitors (Thermo Scientific). Protein concentration was measured using the BCA assay. Equal protein concentrations were loaded per condition. Proteins were separated with Nu-PAGE 4-12% Bis-Tris gels (Invitrogen) using MES x 1 running buffer at 180V constant. Duplicate blots using the same samples were run for NIX detection. Protein were transferred using the dry transfer iBlot2 system (Invitrogen). Equal loading were confirmed with ponceau staining of the membrane. Membranes were incubated in blocking buffer (5% milk in TBST) for 1 hour before incubation with primary antibodies at 4°C over night. After washing 3 times for 5 minutes with TBST membranes were incubated with Li-Cor secondary antibodies for 1 hour at room temperature. A Li-Cor Odyssey infrared scanner was used to detect and quantify proteins. Primary antibodies were as follows: phospho-PDH (ab92696), PDH (ab110334), β-Actin (ab8226), MDH1 (ab180152) and LDHA (ab92903) from Abcam; pan-Cadherin (4068T), Hif-1α (14179S), NIX (12396S) and BNIP3 (44060S) from Cell Signaling Technology; Tom20 (sc-136211) from Santa Cruz Biotechnology.

siRNA Transfection

The siRNA targeting human MDH1, MDH2, LDHA (siGENOME SMART pool siRNA), Hif1α, NIX, BNIP3 (ON-TARGET plus SMART pool siRNA) and a non-targeting control siRNA were purchased from Dharmacon and transfected using Lipofectamine RNAiMAX Transfection Reagent (Invitrogen).

Oxygen Consumption Rates

For respiration assays 293T and SW480 cells grown in attached or detached conditions for 7 days were trypsinised and seeded at a density between 20,000 and 50,000 cells per well in XF96 microplates coated with cell-TAK cell adhesive (Corning). Plates were centrifuged at 200 x g for 1 min to attach cells to surface of the plate. Plates were incubated at 37°C for 15 minutes before XF media (Agilent Seahorse XF) was added. Plates were further incubated at 37°C in a non-CO2 incubator for 30 minutes followed by Mito Stress Test assay (Agilent Seahorse XF). After completion of the assay OCR was normalised to protein content per well using BCA protein assay.

Flow Cytometry

To asses mitochondrial ROS production cells were grown in attached or detached conditions as described above. On the day of the experiment attached and detached cells were trypsinized into single cells and then incubated with MitoSOX Red (Thermo Fisher

Scientific) and DAPI dye in complete DMEM for 30 min at 37°C followed by washing 3 times with PBS and resuspending cells in cold PBS. Cells were analysed by flow cytometry with 488 nm excitation and monitoring fluorescence emitted at 588 nm. DAPI positive cells were excluded from analysis.

To measure mitochondrial mass cells were grown and tripsinized as described above before incubating with Mito Tracker Green (Thermo Fisher Scientific) or NAO (Thermo Fisher Scientific) at 37°C for 30 minutes. After incubation with mitochondrial dyes cells were washed twice with PBS before resuspension in cold PBS. Cells were analysed by flow cytometry with 488 nm excitation and monitoring fluorescence emitted at 530 nm.

For cell viability and cell death measurements cells were trypsinized before incubating with a fixable viability dye eFluor 780 (eBioscience) for 30 minutes at 4°C. Cells were then washed and analysed by monitoring fluorescens emitted at 780 nm after excitation with the 633 nm laser. Cell viability were also measured using DAPI exclusion (Sauvat et al., 2015). Cells were trypsinized and incubated with DAPI for 30 minutes before washing twice with PBS. DAPI negative cells were counted as viable.

For mito-mKeima mitophagy analysis HeLa and A549 cells were transfected with mt-mKeima followed by culturing in attached or detached conditions for 3-4 days. Cells were analysed by flow cytometry as described previously (Lazarou et al., 2015). Fluorescence emitted at 620 were monitored and mt-mKeima in mitochondria in the cytosol were excited by the 405 nm laser while mKeima in mitochondria in lysosomes were excited by the 561 nm laser. Flow cytometry data were analyzed using FlowJo Software (FlowJo, LLC).

Imaging

Fluorescence Microscopy

To determine lipid droplet formation HeLa mito-YFP cells were seeded at 20,000 cells per well in a 33 mm glass bottom FluoroDish (WPI) for attached conditions and the same number of cells were seeded in ultra-low attachment plates for detached conditions. Detached cells were cultured for 7 days and attached cells were cultured in the FluoroDish for 2 days before staining with BODIPY 558/568 C12 (Invitrogen). Imaging was performed using a Zeiss LSM 710 inverted confocal microscope and images were processed using ZEN blue software (Zeiss).

For hypoxia staining cells were cultured in detached conditions for 3 days before staining cells with Image-iT Green Hypoxia Reagent (Thermo Fisher Scientific) according to manufacturer's recommendations. Imaging was performed using the EVOS FL Cell Imaging System (Thermo Fisher scientific) using the brightfield and green fluorescent channels.

Transmission Electron Microscopy

For transmission electron microscopy, cells were fixed by adding warm (to the temperature the cells are maintained) 8% (v/v) formaldehyde (Taab Laboratory Equipment Ltd, Aldermaston, UK) in 0.2M phosphate buffer (PB) pH 7.4 directly to the cell culture medium (1:1) for 15min. The samples were then processed using a Pelco BioWave Pro+ microwave (Ted Pella Inc, Redding, USA) and following a protocol adapted from the National Centre for Microscopy and Imaging Research protocol (Deerinck et al., 2010). See Table S1 for full BioWave program details. Each step was performed in the Biowave, except for the PB and water wash steps, which consisted of two washes on the bench followed by two washes in the Biowave without vacuum (at 250W for 40s). All the chemical incubations were performed in the Biowave for 14min under vacuum in 2min cycles alternating with/without 100W power. The SteadyTemp plate was set to 21°C unless otherwise stated. In brief, the samples were fixed again in 2.5% (v/v) gluteraldehyde (TAAB) / 4% (v/v) formaldehyde in 0.1M PB. The cells were then stained with 2% (v/v) osmium tetroxide (TAAB) / 1.5% (v/v) potassium ferricyanide (Sigma), incubated in 1% (w/v) thiocarbohydrazide (Sigma) with SteadyTemp plate set to 40°C, and further stained with 2% osmium tetroxide in ddH2O (w/v). The cells were then incubated in 1% aqueous uranyl acetate (Agar Scientific, Stansted, UK) with SteadyTemp plate set to 40°C, and then washed in dH_2O with SteadyTemp set to 40°C. Samples were then stained with Walton's lead aspartate with SteadyTemp set to 50°C, and dehydrated in a graded ethanol series (70%, 90%, and 100%, twice each), at 250 W for 40 s without vacuum. Exchange into Durcupan ACM® resin (Sigma) was performed in 50% resin in ethanol, followed by 4 pure Durcupan steps, at 250 W for 3 min, with vacuum cycling (on/off at 30 sec intervals), before embedding at 60°C for 48 h. Blocks were serial sectioned using a UC7 ultramicrotome (Leica Microsystems, Vienna, Austria) and 70nm sections were picked up on 2mm slot copper grids (Gilder Grids Ltd., Grantham, UK). A middle section through the cells was viewed in each condition using a 120 kV Tecnai G2 Spirit transmission electron microscope (FEI Company, Eindhoven, Netherlands) and images of 20-30 cells per condition were captured using an Orius CCD camera (Gatan Inc., Pleasanton, USA). Mitochondrial outer membrane circumference and cristae length were measured manually in Fiji using the measuring tool (Sood et al., 2014). 58 -283 mitochondria were analysed per condition.

In Vivo Experiments

Xenograft Experiment

For tumor growth experiments C57BL16J mice (obtained from The Jackson Laboratory) received bilateral subcutaneous injections with 100 μl of $2.5X10^6$ KPC FC1242 PDAC cells in PBS either grown in glucose or galactose in detached conditions for 4 days. Once tumors were palpable, growth was measured once a week by caliper. Tumor volume was estimated using the formula: length * $width^2/2$

Lung Metastasis Model

$1X10^6$ KPC FC1242 PDAC cells per mouse in 100μl PBS were injected (tail vein) into athymic *nu/nu* mice (obtained from The Jackson Laboratory). After 14 days, lung tissues were collected for histological analysis.

Immunohistochemistry

H&E staining were performed as previously described (Cheung et al., 2016a)

QUANTIFICATION AND STATISTICAL ANALYSIS

Statistical details of experiments can be found in the figure legends. All data are expressed as ± SD or ± SEM as indicated in figure legends. Statistical significance was determined using unpaired Student's t test or Multiple t test. All statistical analysis were carried out in GraphPad Prism7. Significant differences are indicated as follows: *$p \leq 0.05$, **$p \leq 0.01$, ***$p \leq 0.001$, ****$p \leq 0.0001$.

DATA AND CODE AVAILABILITY

Source data for Figure S5E in the paper is available at https://cistrome.shinyapps.io/timer/

Cell Metabolism, Volume *30*

Supplemental Information

Cell Clustering Promotes a Metabolic Switch that Supports Metastatic Colonization

Christiaan F. Labuschagne, Eric C. Cheung, Julianna Blagih, Marie-Charlotte Domart, and Karen H. Vousden

Supplemental Information

Figure S1

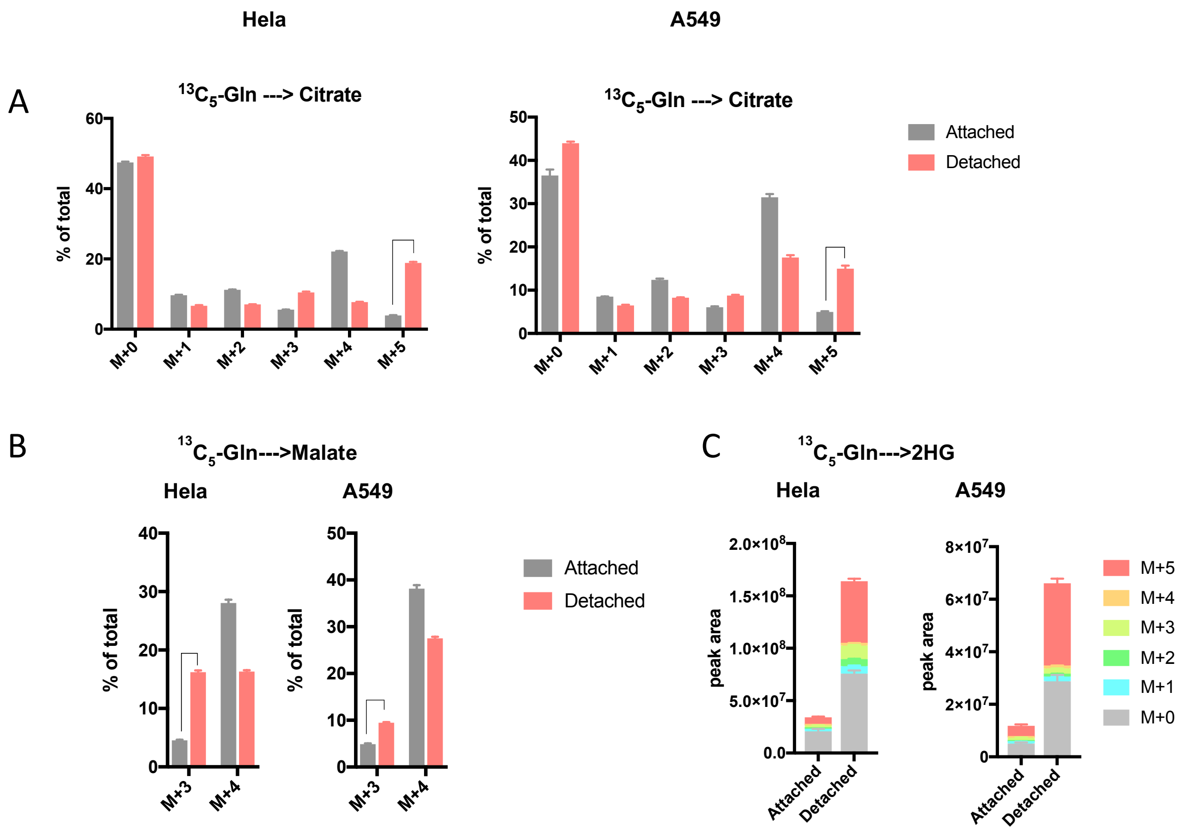

Figure S1. Related to Figure 1.

(A and B) Isotopomer distribution of (A) citrate and (B) malate in attached and detached Hela and A549 cells cultured with $^{13}C_5$-glutamine for 4 hours.

(C) Levels and isotopomer distribution of 2-hydroxyglutarate in attached and detached Hela and A549 cells cultured in the same conditions as (A). Peak area levels are normalized to cell number.

Data are presented as mean ± SD of triplicate cultures of representative experiments.

Figure S2

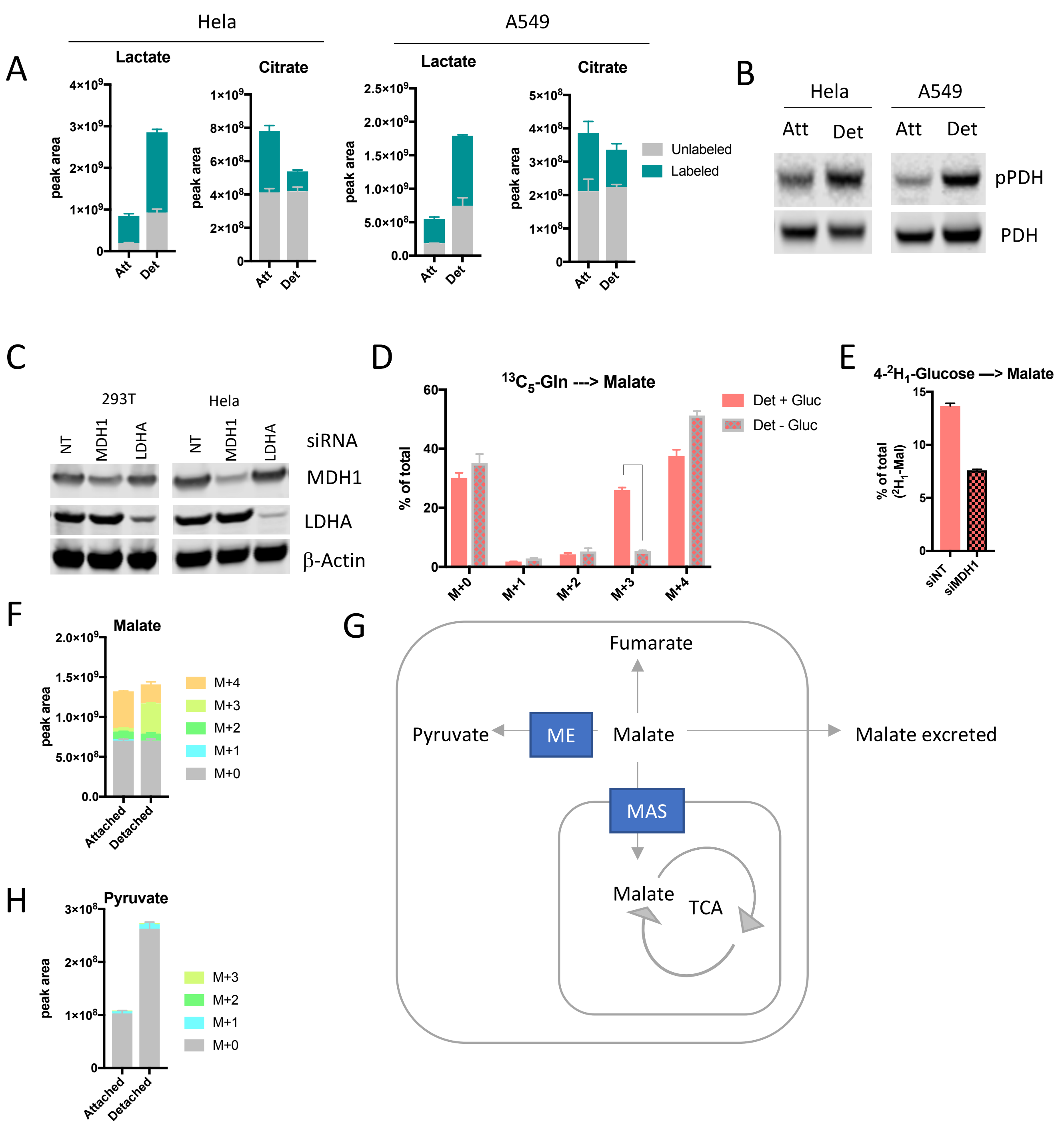

Figure S2. Related to Figure 2

(A) Levels of isotope labelling of glycolytic intermediates of Hela and A549 cells cultured in attached and detached conditions in the presence of $^{13}C_6$-glucose for 4 hours. Peak area levels were normalized to cell number.

(B) Western Blot analysis demonstrating pyruvate dehydrogenase phosphorylation in Hela and A549 attached and detached cells. Equal protein amounts were loaded per condition.

(C) Western blot analysis showing expression levels of MDH1, LDHA and β-Actin after siRNA knock down of MDH1 and LDHA in detached 293T and Hela cells.

(D) Isotopomer distribution of malate in detached 293T cells labelled with $^{13}C_5$-glutamine for 4 hours in the presence or absence of glucose.

(E) Levels of deuterium labelled malate from 4-$^2H_1$-glucose labelling in detached A549 cells following siRNA knock down of MDH1.

(F) Levels and isotopomer distribution of malate in attached and detached 293T cells labelled with $^{13}C_5$-glutamine for 4 hours.

(G) A schematic representation of the fate of cytosolic produced malate in detached cells.

(H) Levels and isotopomer distribution of pyruvate in attached and detached 293T cells labelled with $^{13}C_5$-glutamine for 4 hours.

(A, D, E, F and H) Data are presented as mean $\pm$ SD of triplicate cultures of representative experiments.

Figure S3

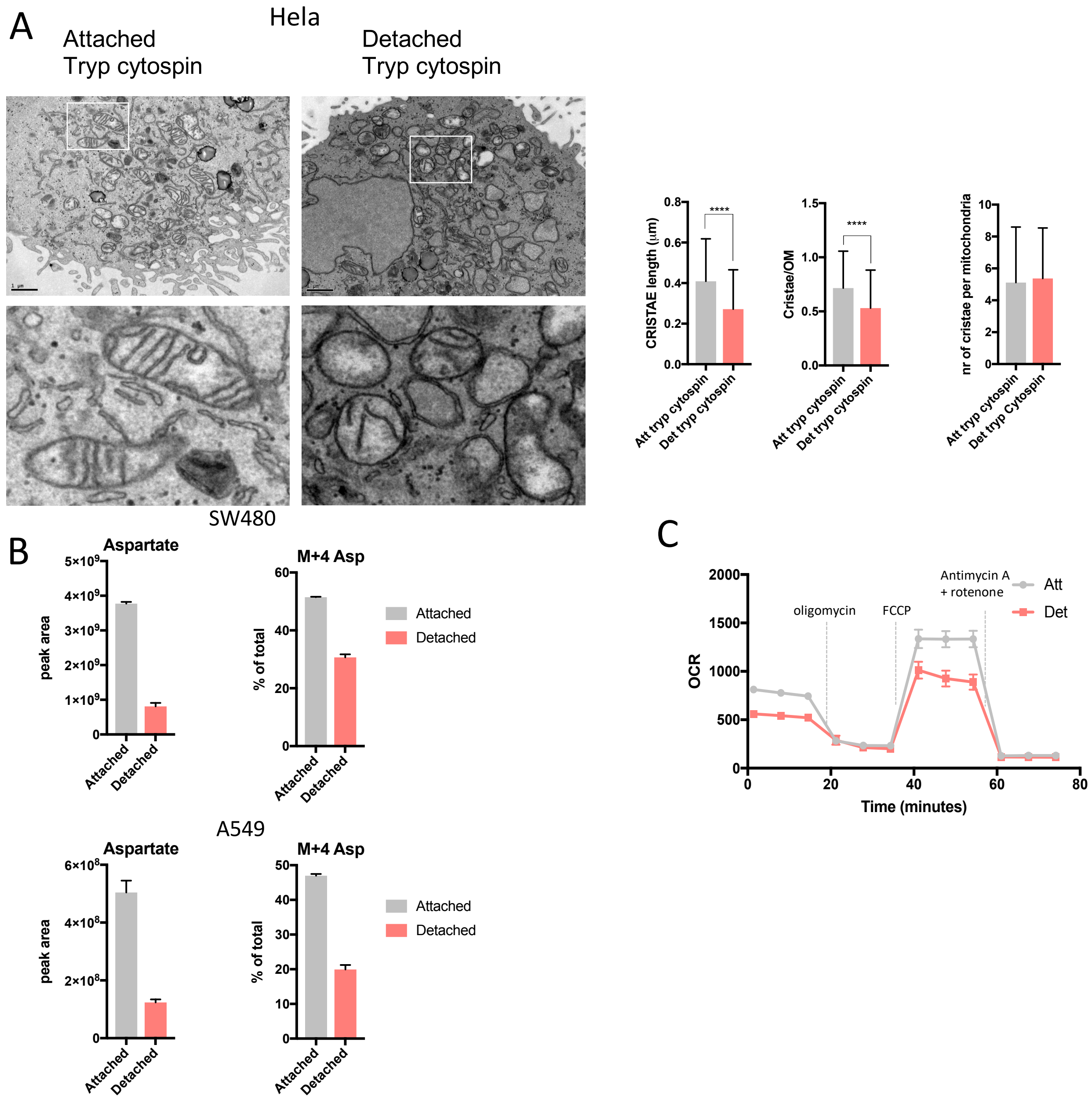

Figure S3. Related to Figure 3

(A) Representative transmission electron microscopy images of mitochondria of attached and detached cells trypsinised into single cells before cytospin to attach cells to glass bottom plates. Graphs present quantified cristae length, cristae/outer membrane ratio and number of cristae per mitochondria (Att n= 136 mitochondria and Det n=250 mitochondria). ****$p \leq 0.0001$, unpaired t test.

(B) Intracellular levels of unlabelled and M+4 labelled aspartate from $^{13}C_5$-glutamine in attached and detached SW480 and A549 cells. Peak area levels are normalized to cell number.

(C) Oxygen consumption rate profiles of SW480 cells cultured in attached or detached conditions. Dotted lines show incubation of cells in the presence of indicated mitochondrial inhibitors. Data are normalised to protein content.

(A, B and C) Data are presented as mean ± SD of triplicate cultures of representative experiments.

Figure S4

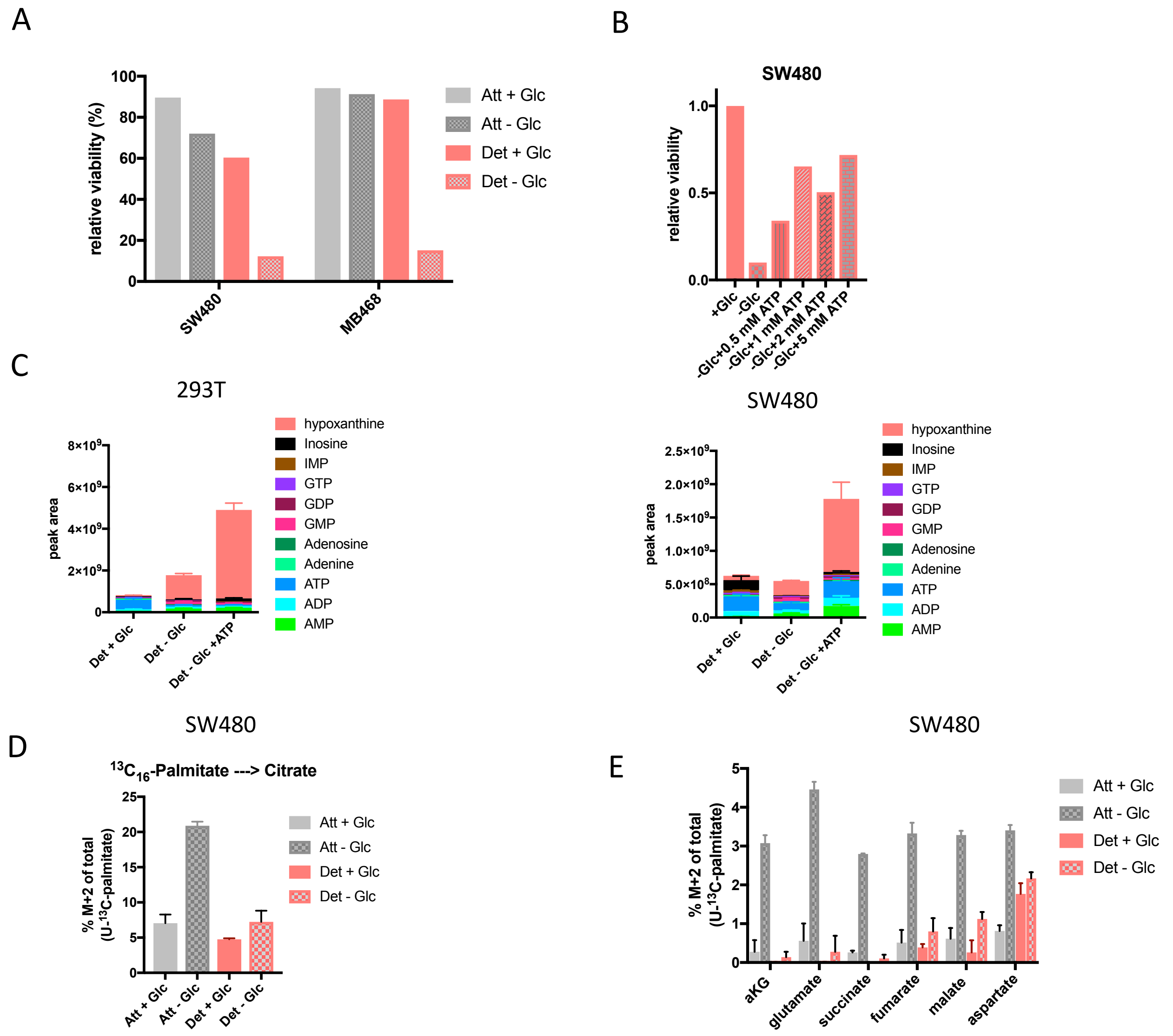

Figure S4. Related to Figure 4

(A) Cell viability of SW480 and MB468 cells cultured in attached and detached conditions in the presence or absence of glucose. Cells were cultured in attached or detached conditions for 7 days in the presence of glucose before glucose starvation for 24 hours followed by staining with eFluor 780 viability dye and analysis by flow cytometry.

(B) Cell viability of SW480 cells as measured in (A) in cells cultured with glucose or without glucose plus indicated ATP concentrations.

(C) Intracellular nucleotide levels of detached 293T and SW480 cells cultured with or without glucose plus 5 mM ATP. Peak area levels are normalised to cell number.

(D and E) Levels of M+2 citrate (D) and other TCA cycle intermediates (E) originating from $^{13}C_{16}$-palmitate in SW480 attached and detached cells cultured with or without glucose. Cells were cultured as in (A) before addition of labelled palmitate and removal of glucose for 4 hours.

(C-E) Data are presented as mean ± SD of triplicate cultures of representative experiments.

Figure S5

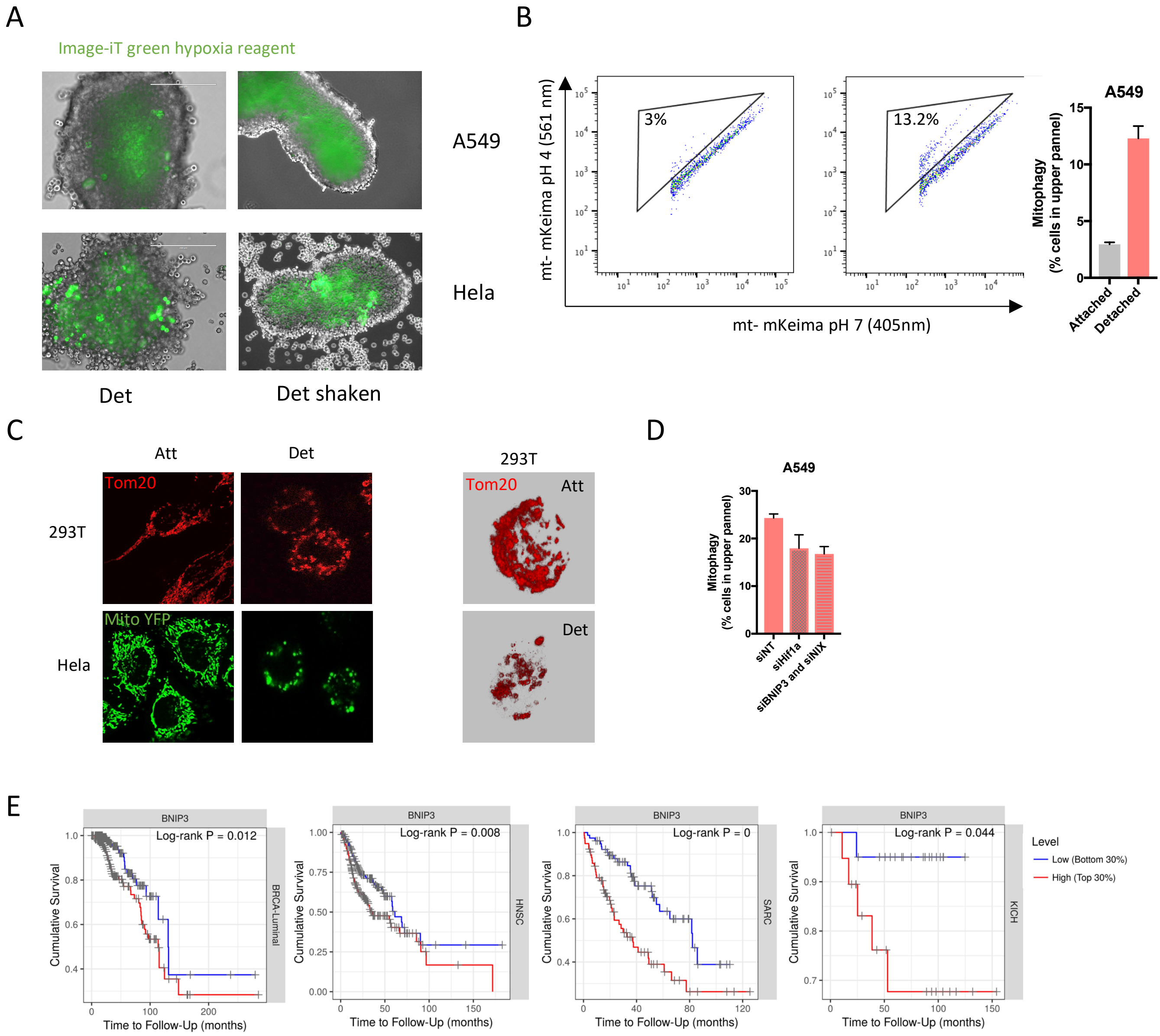

Figure S5. Related to Figure 5

(A) Image-iT green hypoxia reagent staining of detached A549 and Hela cells cultured in static (left panel) and shaken (right panel) conditions. Cells were cultured in detached conditions for 3 days before live cell staining was performed.

(B) A549 cells were transfected with mitochondrial targeted-mkeima and cultured in attached or detached conditions for 3 days before flow cytometry analysis. Mitochondria in cytosol (pH 7) are represented by mkeima with excitation of 405 nm while mitochondria in lysosomes (pH 4) are represented by mkeima with excitation of 561 nm. Mitophagy is reflected by the percentage of cells in the upper panel.

(C) Confocal microscopy images of attached and detached 293T and Hela cells. 293T cells were grown in attached or detached conditions for 7 days before cells were fixed followed by immunostaining with Tom20 antibody. Hela cells expressing Mito YFP were cultured in attached or detached conditions for 7 days before imaging.

(D) Mitochondrial targeted-mkeima expressing A549 cells were cultured in detached conditions in the presence of non-targeting, Hif1α or BNIP3 + NIX siRNA and analysed by flow cytometry for mitophagy as in (B).
(E) Patient survival data of tumors expressing high and low levels of BNIP3 in indicated cancers types.
(B and D) Data are presented as mean $\pm$ SD of triplicate cultures of a representative experiment.

Figure S6

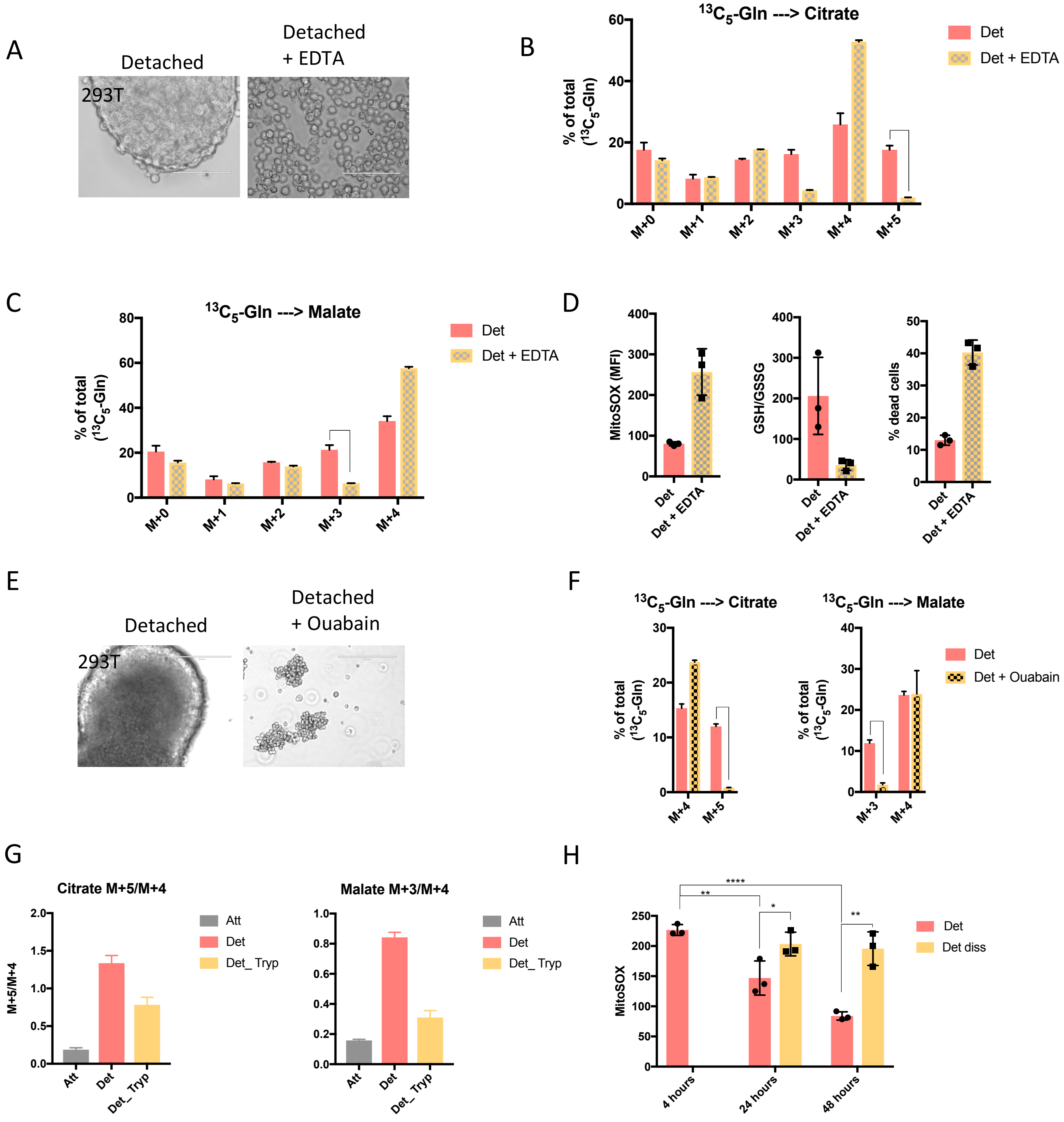

Figure S6. Related to Figure 6

(A) Representative images of 293T cells treated with 2mM EDTA to prevent clustering after matrix detachment. Cells were cultured for 48 hours in detached conditions in the presence of EDTA. Scale bars are 100 μm.

(B and C) Isotopomer distribution of citrate (B) and malate (C) in 293T cells grown in detached conditions with or without EDTA.

(D) Preventing cell clustering after matrix detachment induce an increase in mitochondrial ROS production, oxidative stress and cell death. 293T cells were cultured in detached conditions with or without EDTA for 3 days before GSH/GSSG, mitochondrial ROS and cell viability analysis.

(E) Representative images of 293T cells treated with 0.5 μM Ouabain to prevent clustering after matrix detachment. Cells were cultured for 3 days in detached conditions in the presence of Ouabain. Scale bars are 200 μm.

(F) M+4 and M+5 isotopologues of citrate (left panel) and M+3 and M+4 isotopologues of malate (right panel) originating from $^{13}C_5$-glutamine in 293T cells cultured in detached conditions with and without 0.5 μM Ouabain.

(G) M+5/M+4 isotopologues of citrate (left panel) and M+3/M+4 of malate (right panel) originating from $^{13}C_5$-glutamine in 293T cells cultured in attached conditions, detached conditions and detached conditions before dissociation into single cells (Det diss).

(H) Levels of mitochondrial ROS production in 293T cells cultured in detached conditions and detached conditions before dissociation into single cells for indicated times. Mitochondrial ROS was measured by staining cells with MitoSOX Red before analysis by flow cytometry.

(B,C,D,F,G and H) Data are represented as mean $\pm$ SD of triplicate cultures of a representative experiment.

*$p \leq 0.05$, **$p \leq 0.01$, ****$p \leq 0.0001$ Unpaired t test.

Figure S7

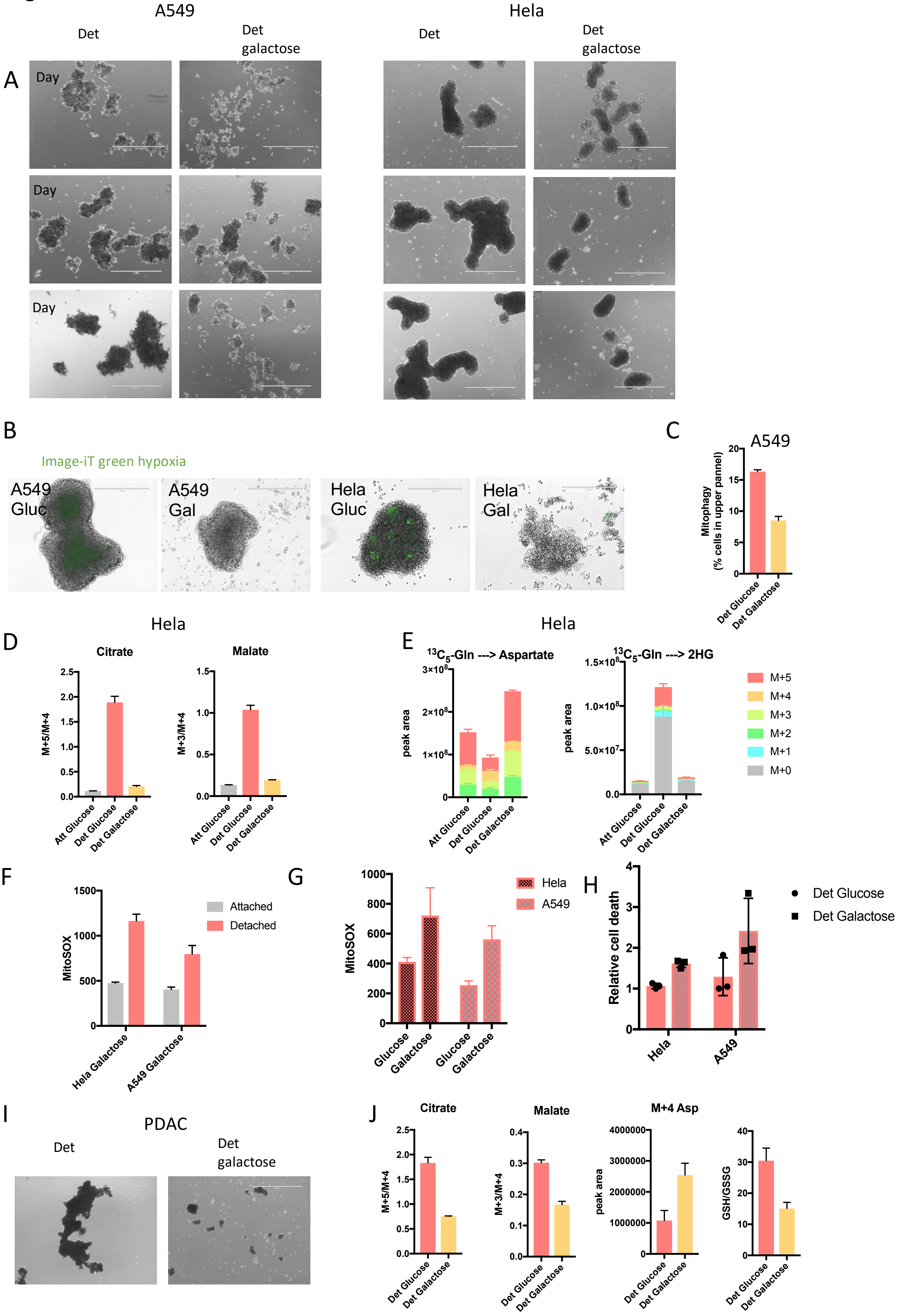

A549
Hela
Det
Det galactose
A
Day
Day
Day
B
Image-iT green hypoxia
A549 Gluc
A549 Gal
Hela Gluc
Hela Gal
C
A549
Mitophagy (% cells in upper pannel)
Det Glucose
Det Galactose
D
Hela
Citrate
Malate
M+5/M+4
M+3/M+4
Att Glucose
Det Glucose
Det Galactose
E
Hela
$^{13}C_5$-Gln ---> Aspartate
$^{13}C_5$-Gln ---> 2HG
peak area
M+5
M+4
M+3
M+2
M+1
M+0
F
MitoSOX
Attached
Detached
Hela Galactose
A549 Galactose
G
Hela
A549
Glucose
Galactose
H
Relative cell death
Det Glucose
Det Galactose
Hela
A549
I
PDAC
Det
Det galactose
J
Citrate
Malate
M+4 Asp
GSH/GSSG

Figure S7. Related to Figure 7

(A) Representative images of detached A549 and Hela cells grown in glucose or galactose for 3 days. Scale bars are 1000 μm.

(B) Image-iT green hypoxia reagent staining of detached A549 and Hela cells grown in glucose or galactose for 3 days before live cell staining was performed. Scale bars are 400 μm.

(C) Mitochondrial targeted-mkeima expressing Hela cells were cultured in detached conditions in glucose or galactose and analysed by flow cytometry for mitophagy.

(D) $^{13}C_5$-labeled glutamine derived M+5/M+4 citrate and M+3/M+4 malate of Hela cells grown in attached and detached conditions with media containing either glucose or galactose.

(E) Levels and isotopomer distribution of intracellular aspartate and 2-HG of Hela cells cultured in the same conditions as (C). Peak area levels are normalized to cell number.

(F) Mitochondrial ROS analysis using MitoSOX Red dye in attached and detached Hela and A549 cells grown in galactose. Cells were cultured in attached conditions or detached conditions with galactose for indicated times before MitoSOX Red staining and analysis. MitoSOX fluorescence was analysed by flow cytometry.

(G) Mitochondrial ROS analysis using MitoSOX Red dye in detached Hela and A549 cells grown in glucose or galactose.

(H) Fraction of dead Hela and A549 cells grown in detached conditions with either glucose or galactose.

(I) Representative image of PDAC cells grown detached in glucose or galactose. Scale bars are 1000 μm.

(J) $^{13}C_5$-labeled glutamine derived M+5/M+4 citrate, M+3/M+4 malate, M+4 Aspartate and GSH/GSSG of PDAC cells grown in detached conditions with media containing either glucose or galactose.

(C, D, E, F, G, H and J) Data are presented as mean ± SD of triplicate cultures of a representative experiment.

Supplemental Table

Table S1

Description	Step#	Time (min)	Time (sec)	Power (Watts)	SteadyTemp temperature (ºC)	Vacuum cycle vent time (sec)	Vacuum cycle vacuum time (sec)	Vacuum set point (inch Hg)	User Prompt (1 = YES, 0 = NO)	Vacuum OFF (1 = no vacuum, 0 = vacuum)	Vacuum cycle (1 = ON,0=OFF)	Vacuum ON (1 = vacuum, 0 = no vacuum)
BENCH STEP Rinse in 0.1M PB	1	0	0	0	21	0	0	0	1	1	0	0
BENCH STEP Rinse in 0.1M PB	2	0	0	0	21	0	0	0	1	1	0	0
Rinse in 0.1M PB	3	0	40	250	21	0	0	0	1	1	0	0
Rinse in 0.1M PB	4	0	40	250	21	0	0	0	1	1	0	0
Osmium ON	5	2	0	100	21	0	0	20	1	0	0	1
Osmium OFF	6	2	0	0	21	0	0	20	0	0	0	1
Osmium ON	7	2	0	100	21	0	0	20	0	0	0	1
Osmium OFF	8	2	0	0	21	0	0	20	0	0	0	1
Osmium ON	9	2	0	100	21	0	0	20	0	0	0	1
Osmium OFF	10	2	0	0	21	0	0	20	0	0	0	1
Osmium ON	11	2	0	100	21	0	0	20	0	0	0	1
BENCH STEP Rinse in water	12	0	0	0	21	0	0	0	1	1	0	0
BENCH STEP Rinse in water	13	0	0	0	21	0	0	0	1	1	0	0
Rinse in water	14	0	40	250	21	0	0	0	1	1	0	0
Rinse in water	15	0	40	250	21	0	0	0	1	1	0	0
Thiocarbohydrazide ON	16	2	0	100	40	0	0	20	1	0	0	1
Thiocarbohydrazide OFF	17	2	0	0	40	0	0	20	0	0	0	1
Thiocarbohydrazide ON	18	2	0	100	40	0	0	20	0	0	0	1
Thiocarbohydrazide OFF	19	2	0	0	40	0	0	20	0	0	0	1
Thiocarbohydrazide ON	20	2	0	100	40	0	0	20	0	0	0	1
Thiocarbohydrazide OFF	21	2	0	0	40	0	0	20	0	0	0	1
Thiocarbohydrazide ON	22	2	0	100	40	0	0	20	0	0	0	1
BENCH STEP Rinse in water	23	0	0	0	21	0	0	0	1	1	0	0
BENCH STEP Rinse in water	24	0	0	0	21	0	0	0	1	1	0	0
Rinse in water	25	0	40	250	21	0	0	0	1	1	0	0
Rinse in water	26	0	40	250	21	0	0	0	1	1	0	0
Osmium ON	27	2	0	100	21	0	0	20	1	0	0	1
Osmium OFF	28	2	0	0	21	0	0	20	0	0	0	1
Osmium ON	29	2	0	100	21	0	0	20	0	0	0	1
Osmium OFF	30	2	0	0	21	0	0	20	0	0	0	1
Osmium ON	31	2	0	100	21	0	0	20	0	0	0	1
Osmium OFF	32	2	0	0	21	0	0	20	0	0	0	1
Osmium ON	33	2	0	100	21	0	0	20	0	0	0	1
BENCH STEP Rinse in water	34	0	0	0	21	0	0	0	1	1	0	0
BENCH STEP Rinse in water	35	0	0	0	21	0	0	0	1	1	0	0
Rinse in water	36	0	40	250	21	0	0	0	1	1	0	0
Rinse in water	37	0	40	250	21	0	0	0	1	1	0	0
Uranyl acetate ON	38	2	0	100	40	0	0	20	1	0	0	1
Uranyl acetate OFF	39	2	0	0	40	0	0	20	0	0	0	1
Uranyl acetate ON	40	2	0	100	40	0	0	20	0	0	0	1
Uranyl acetate OFF	41	2	0	0	40	0	0	20	0	0	0	1
Uranlyl acetate ON	42	2	0	100	40	0	0	20	0	0	0	1
Uranyl acetate OFF	43	2	0	0	40	0	0	20	0	0	0	1
Uranyl acetate ON	44	2	0	100	40	0	0	20	0	0	0	1
BENCH STEP Rinse in water	45	0	0	0	40	0	0	0	1	1	0	0
BENCH STEP Rinse in water	46	0	0	0	40	0	0	0	1	1	0	0
Rinse in water	47	0	45	250	40	0	0	0	1	1	0	0
Rinse in water	48	0	45	250	40	0	0	0	1	1	0	0
Lead aspartate ON	49	2	0	100	50	0	0	20	1	0	0	1
Lead aspartate OFF	50	2	0	0	50	0	0	20	0	0	0	1
Lead aspartate ON	51	2	0	100	50	0	0	20	0	0	0	1
Lead aspartate OFF	52	2	0	0	50	0	0	20	0	0	0	1
Lead aspartate ON	53	2	0	100	50	0	0	20	0	0	0	1
Lead aspartate OFF	54	2	0	0	50	0	0	20	0	0	0	1
Lead aspartate ON	55	2	0	100	50	0	0	20	0	0	0	1
BENCH STEP Rinse in water	56	0	0	0	21	0	0	0	1	1	0	0
BENCH STEP Rinse in water	57	0	0	0	21	0	0	0	1	1	0	0
Rinse in water	58	0	45	250	21	0	0	0	1	1	0	0
Rinse in water	59	0	45	250	21	0	0	0	1	1	0	0
70% Ethanol ON	60	0	40	250	21	0	0	0	1	1	0	0
70% Ethanol ON	61	0	40	250	21	0	0	0	1	1	0	0
90% Ethanol ON	62	0	40	250	21	0	0	0	1	1	0	0
90% Ethanol ON	63	0	40	250	21	0	0	0	1	1	0	0
100% Ethanol ON	64	0	40	250	21	0	0	0	1	1	0	0
100% Ethanol ON	65	0	40	250	21	0	0	0	1	1	0	0
50% Resin ON	66	3	0	250	21	30	30	20	1	0	1	0
100% Resin ON	67	3	0	250	21	30	30	20	1	0	1	0
100% Resin ON	68	3	0	250	21	30	30	20	1	0	1	0
100% Resin ON	69	3	0	250	21	30	30	20	1	0	1	0
100% Resin ON	70	3	0	250	21	30	30	20	1	0	1	0
TURN SYSTEM OFF	71	0	0	0	21	0	0	0	0	1	0	0

Table S1. Related to STAR Methods. BioWave program details
